# Transcriptional regulation of the proto‐oncogene *Zfp521* by SPI1 (PU.1) and HOXC13

**DOI:** 10.1002/dvg.22963

**Published:** 2016-08-29

**Authors:** Ming Yu, Salma Al‐Dallal, Latifa Al‐Haj, Shiraj Panjwani, Akina S. McCartney, Sarah M. Edwards, Pooja Manjunath, Catherine Walker, Alexander Awgulewitsch, Kathryn E. Hentges

**Affiliations:** ^1^ Faculty of Life SciencesUniversity of ManchesterManchesterM13 9PTUK; ^2^ The Key Laboratory of Stem Cell and Regenerative MedicineInstitute of Molecular and Clinical Medicine, Kunming Medical UniversityKunmingYunnan Province650500People's Republic of China; ^3^ Molecular Biomedicine Program, Program in Biomolecular ResearchKing Faisal Specialist Hospital and Research CenterRiyadh11211Saudi Arabia; ^4^ Department of MedicineMedical University of South CarolinaCharlestonSC29425

**Keywords:** B‐cell leukemia, B‐cell differentiation, AKXD mice, ZNF521, Evi3

## Abstract

The mouse zinc‐finger gene *Zfp521* (also known as ecotropic viral insertion site 3; *Evi3*; and *ZNF521* in humans) has been identified as a B‐cell proto‐oncogene, causing leukemia in mice following retroviral insertions in its promoter region that drive *Zfp521* over‐expression. Furthermore, *ZNF521* is expressed in human hematopoietic cells, and translocations between *ZNF521* and *PAX5* are associated with pediatric acute lymphoblastic leukemia. However, the regulatory factors that control *Zfp521* expression directly have not been characterized. Here we demonstrate that the transcription factors SPI1 (PU.1) and HOXC13 synergistically regulate *Zfp521* expression, and identify the regions of the *Zfp521* promoter required for this transcriptional activity. We also show that SPI1 and HOXC13 activate *Zfp521* in a dose‐dependent manner. Our data support a role for this regulatory mechanism *in vivo*, as transgenic mice over‐expressing *Hoxc13* in the fetal liver show a strong correlation between *Hoxc13* expression levels and *Zfp521* expression. Overall these experiments provide insights into the regulation of *Zfp521* expression in a nononcogenic context. The identification of transcription factors capable of activating *Zfp521* provides a foundation for further investigation of the regulatory mechanisms involved in ZFP521‐driven cell differentiation processes and diseases linked to *Zfp521* mis‐expression.

## 1 Introduction

Alterations in gene expression during lymphocyte differentiation can lead to malignancies including leukemia and lymphoma. Retroviral insertions that cause up‐regulation of *Zinc finger protein 521* (*Zfp521*) or its paralogue, *Zinc finger protein 423* (*Zfp423*), are associated with B‐cell leukemia in mice (Hentges et al., 2005; Warming et al., 2003, 2004). Links between *ZNF521* (the human orthologue of *Zfp521*) and human leukemia also exist, as a translocation that generates a chimaeric fusion protein of ZNF521 and PAX5 has been found in paediatric acute lymphoblastic leukemia (Mullighan et al., 2007). Retroviral insertions at the *Zfp521* locus also promote the formation of B‐cell acute lymphoblastic leukemia (B‐ALL) in mice expressing the chimaeric oncogenic fusion protein E2A‐hepatic leukemia factor (E2A‐HLF), and *ZNF521* overexpression is found in patients with translocations generating E2A‐HLF fusion proteins (Yamasaki et al., 2010). Recent investigations have established a role for *Zfp521* in B‐cell differentiation, mediated through an interaction with the B‐cell transcription factor EBF (Hentges et al., 2005; Hiratsuka et al., 2015; Mega et al., 2011). Additional functions for ZFP521 and its paralogue ZFP423 have been identified, demonstrating that these multiple zinc‐finger proteins participate in cell proliferation and differentiation events critical for the formation of a diverse set of cell types. An important role for ZFP521 in cell differentiation events has been documented for neural cells (Han et al., 2012; Hashemi et al., 2016; Kamiya et al., 2011; Lobo et al., 2006; Ohkubo et al., 2014; Tang et al., 2015), erythrocytes (Matsubara et al., 2009), chondrocytes (Correa et al., 2010; Hesse et al., 2010; Kiviranta et al., 2013; Park and Kim, 2013) and adipocytes (Kang et al., 2012). Similarly, ZFP423 has been identified as a key factor in adipocyte differentiation (Addison et al., 2014; Gupta et al., 2010; Gupta et al., 2012; Hiratsuka et al., 2015), in addition to being required for cerebellar development (Warming et al., 2006). The protein domains and interaction partners of ZFP521 and ZFP423 required in different cell types varies (Correa et al., 2010; Hesse et al., 2010; Kamiya et al., 2011; Mega et al., 2011; Spina et al., 2013), suggesting multiple mechanisms by which these large zinc‐finger proteins can regulate cellular activities. Despite the emerging evidence that ZFP521 and ZFP423 are important factors in determining cell fate, information regarding the regulation of their expression in lymphocytes has been limited to the viral‐mediated over‐expression of these genes seen in B‐cell leukemia. Therefore, we sought to identify factors that directly regulate *Zfp521* expression during B‐cell differentiation.

B‐cell differentiation involves a complex cascade of transcription factor activity leading to specific patterns of gene expression in cells at various stages of differentiation (Busslinger, 2004; Dias et al., 2008; Medina & Singh, 2005; Northrup & Allman, 2008; Nutt & Kee, 2007; Singh et al., 2005). The ETS‐family transcription factor SPI1 (also referred to as PU.1) functions in directing cell fate during haematopoiesis (Oikawa et al., 1999). Along with a role in myeloid lineage commitment, SPI1 is needed for the generation of lymphoid progenitors during B‐cell differentiation, and mice lacking *Spi1* fail to form B‐cells (Scott et al., 1994). SPI1 functions early in the process of B‐cell development to specify lymphoid progenitors by activating the expression of genes such as the IL7 receptor, which are essential for B‐cell differentiation (DeKoter et al., 2002). SPI1 binds to DNA as a monomer through its ETS‐domain (Kodandapani et al., 1996), and also acts cooperatively with various other DNA binding proteins, including other ETS family transcription factors, to activate transcription of target genes (Li et al., 2000).

The homeodomain is a DNA‐binding protein domain present in many developmentally important transcription factors. Several studies have revealed roles for *Hox* genes in normal haematopoiesis and in haematological malignancies such as leukemia (reviewed in (Argiropoulos and Humphries, 2007)). *Hox* genes have a variety of roles in B‐cell differentiation and function. For example, over expression of human *HOXB3* in mouse bone marrow results in a decrease in the total number of B220+ B‐cells (Sauvageau et al., 1997). Conversely, deletion of *Hoxb3* in the bone marrow of adult mice also inhibits B‐cell differentiation, with knock out animals showing a reduced number of pro‐B cells (Ko et al., 2007). Deletion of the homeobox gene *Hoxa9* causes a reduction in the number of lymphocytes present in the spleen of adult mice, due to defects in the specification of committed B‐lymphocyte progenitor cells in the bone marrow (Lawrence et al., 1997). In addition to these roles in B‐cell differentiation, *Hox* genes are also associated with leukemia. For example, genes in the *Hoxa* cluster are frequent targets of murine leukemia retroviral insertions (Bijl et al., 2005). *HOX* gene over‐expression is also a common feature of human lymphoid malignancies, indicating that dysregulation of *HOX* genes may be an important mechanism for leukemic transformation (Argiropoulos and Humphries, 2007).

The phenotypes of mouse mutants of both *Spi1* and *Hox* genes reveal roles for these factors in B‐cell development and differentiation. The expression profile of *Zfp521*, and its over‐expression in mouse B‐cell leukemia, suggests that appropriate regulation of *Zfp521* is important for B‐cell function. We therefore sought to identify transcription factors required for expression of the *Zfp521* gene, finding that SPI1 and HOXC13 synergistically regulate *Zfp521* expression in a dose‐dependent manner. Furthermore, transgenic mice over‐expressing *Hoxc13* also have increased *Zfp521* expression in the fetal liver, the site of B‐cell differentiation during development. Further studies are needed to examine whether SPI1 and HOXC13 regulate *Zfp521* in additional cell types, and whether alterations in the activity of these regulatory factors contributes to B‐cell leukemogenesis.

## 2 Results

### 2.1 Evolutionary analysis of Zfp521 and Zfp423

**Figure 1:**
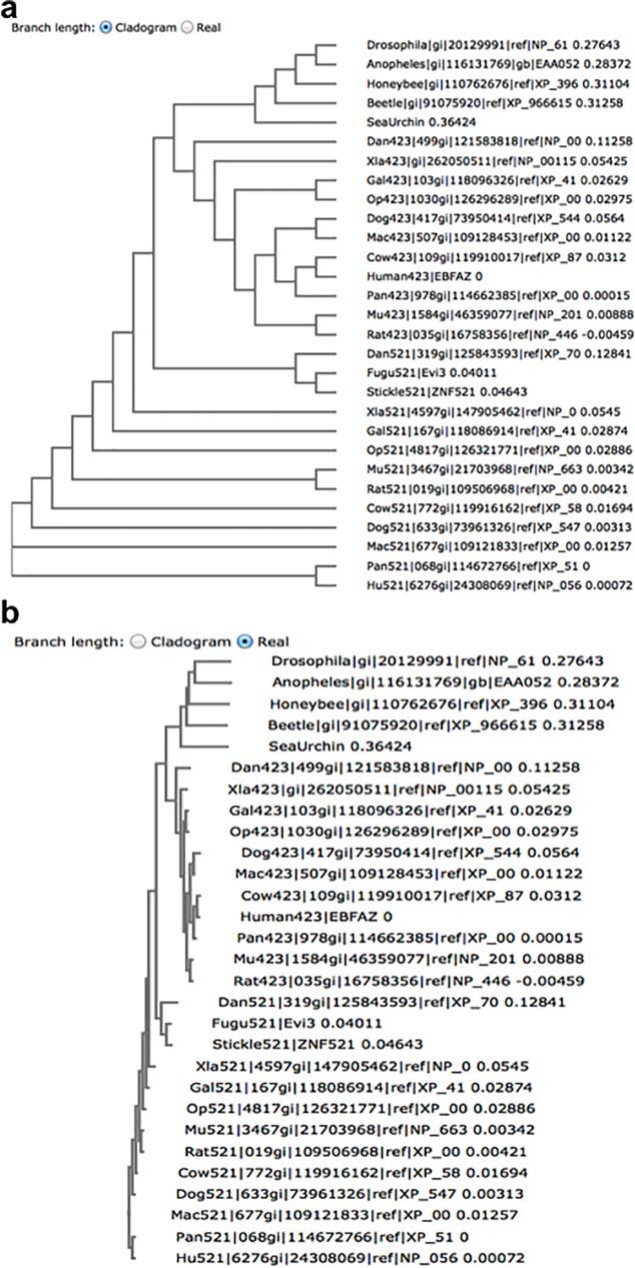
Phylogenetic analysis of ZNF521 and ZNF423. Trees demonstrating the relationship between ZNF521 and ZNF423 protein sequences in a variety of species. a: tree branch length by cladogram. b: tree real branch length. Dan = *Danio rerio*, Fugu = *Takifugu rubripes*, Gal = *Gallus gallus*, Hu = *Homo sapiens*, Mac = *Macaca mulatta*, Mu = *Mus musculus*, Op = *Monodelphis domestica*, Pan = *Pan troglodytes*, Rat = *Rattus norvegicus*, Stickle = Stickleback (*Gasterosteus aculeatus*), Xla = *Xenopus laevis*. Other species as named on tree. 521 = ZNF521/Zfp521, 423= ZNF423/Zfp423

The close protein sequence similarity between ZFP521 and ZFP423 suggests that the genes encoding these proteins arose due to a gene duplication event during evolution. We identified orthologues of ZFP521 and ZFP423 based on protein sequences from genomes of both invertebrate and vertebrate organisms (Supporting Information Table 1), and assembled a phylogenetic tree. We found that the vertebrates analysed have both ZFP521‐ and ZFP423‐related protein sequences (Figure 1a,b), while organisms such as insects have only one single protein sequence that is equally related to ZFP521 and ZFP423. This finding suggests that vertebrates have retained both genes following a duplication event. A reason for paraloguous genes to be retained after duplication events is that the paralogoues have specialized such that the two paralogues no longer have conserved functions or conserved expression patterns. Given that both *Zfp521* and *Zfp423* cause leukemia in mice when over‐expressed (Hentges et al., 2005; Warming et al., 2003; Warming et al., 2004), they may both retain similar functions in lymphocytes. However, their expression patterns in B‐cells have diverged (Warming et al., 2003; Warming et al., 2004), providing an explanation for the retention of both paralogues in vertebrate genomes. Due to the noted expression of *Zfp521* in B‐cells during differentiation (Hiratsuka et al., 2015; Warming et al., 2003), and links between *Zfp521* over‐expression and B‐cell leukemia (Hentges et al., 2005; Warming et al., 2003), we sought to identify transcriptional regulators of *Zfp521*.

### 2.2 *Zfp521* promoter analysis

**Figure 2:**
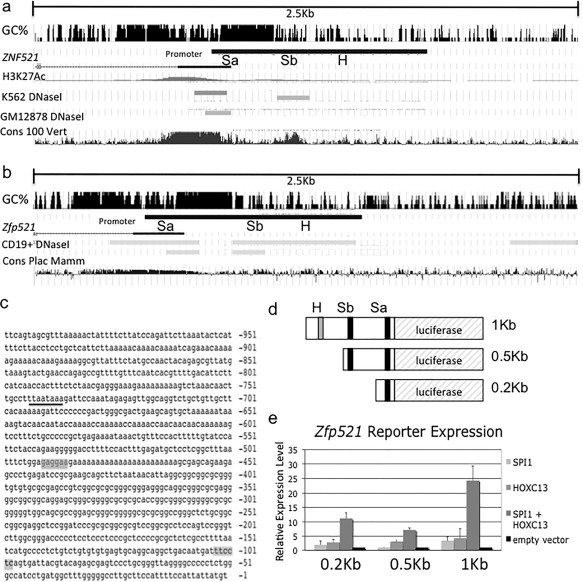
The human and mouse *ZNF521/Zfp521* promoter regions contain SPI1 and HOXC13 predicted binding sites. a: Human chromosome 18q11.2. Scale bar = 2.5 Kb. The GC% in 5‐base windows is shown at the top. The human *ZNF521* promoter region used in this study is shown as a black box labelled promoter. *ZNF521* exon 1 is annotated. H3K27Ac marks, DNaseI hypersensitivity hot spots in K562 erythroleukemia cells, and DNaseI hypersensitivity hot spots in GM12878 lymphocyte cells are shown. Sequence conservation among 100 vertebrates is shown at the bottom. The locations of the predicted SPI1 (Sa and Sb) and HOXC13 (H) binding sites are labeled. Sites Sa and Sb overlap with K562 DNase I hypersensitivity hot spots. b: Mouse chromosome 18qA1. Scale bar = 2.5Kb. The GC% in 5‐base windows is shown at the top. The mouse *Zfp521* region used in this study and in experimental constructs is shown as a black box labeled promoter. *Zfp521* exon is annotated. DNaseI hypersensitivity hotspots in CD19+ B‐cells are annotated. Sequence conservation among placental mammals is shown at the bottom. The locations of the predicted SPI1 (Sa and Sb) and HOXC13 (H) binding sites are labelled. Sites Sa and Sb overlap with CD19+ B‐cell DNase I hypersensitivity hot spots. c: The mouse *Zfp521* promoter sequence. Predicted SPI1 binding sites are shown in gray, and the predicted HOXC13 binding site is underlined. d: *Zfp521* reporter constructs used in transfection assays. Gray box represents predicted HOXC13 binding site, black boxes represent SPI1 binding sites. e: Reporter assay measuring *Zfp521* promoter activation following HEK293 cell transfection with SPI1 (light gray bars), HOXC13 (medium gray bars), or SPI1 and HOXC13 cotransfection (dark gray bars). Relative expression levels are shown as normalized to transfection with reporter alone (black bars). Cotransfection of SPI1 and HOXC13 shows a statistically significant activation of the 1Kb reporter construct when compared to vector control (t‐test; p < 0.05). Error bars represent standard deviation of 3 independent transfections, each with three technical replicates

In order to identify transcriptional regulators of *ZNF521*, an analysis of the promoter region was performed. We identified a 1Kb region upstream of the human *ZNF521* gene transcription start site as annotated in the UCSC genome browser (Figure 2a), and the corresponding region 1Kb upstream of the annotated mouse *Zfp521* transcriptional start site from the UCSC genome browser (Figure 2b). DNaseI hypersensitivity sites are present near the *ZNF521* transcriptional start site, which were experimentally identified from GM12878 and K562 lymphocyte cells (Ho & Crabtree, 2010). The *Zfp521* promoter region shows hypersenstivity to DNaseI cleavage in CD19+ B‐cells isolated from an 8‐week old mouse (Sabo et al., 2006), suggesting the *Zfp521* promoter region has transcriptional activity in B‐cells. Both the mouse and human promoter regions lack a consensus TATA box, but instead have a GC‐rich region near the transcriptional start site (Figure 2a,b). There is a region of high conservation amongst 100 vertebrates in the *ZNF521* promoter region overlapping with a K562 DNase I hypersensitivity hot spot (Figure 2a), although conservation of the mouse *Zfp521* promoter region amongst placental mammals does not show a similar region of high conservation (Figure 2b).

Sequence alignments between the mouse and human *ZNF521* promoters revealed the presence of two conserved binding sites for the transcription factor SPI1, a known B‐cell transcription factor (Figure 2c, gray boxes). We defined the proximal site as SPI1a and the distal site as SPI1b. Both SPI1 sites overlap with regions of DNase I hypersensitivity, suggesting these promoter regions are bound by regulatory proteins in lymphocytes and leukemia cells. A similar analysis of the *ZNF423* gene promoter did not reveal any SPI1 binding sequences (data not shown). In addition to the predicted SPI1 binding sites in the *ZNF521* promoter, we also found a predicted conserved binding site for HOXC13 distal to the predicted SPI1 binding sites (Figure 2c, underline). HOXC13 has been demonstrated to bind the ETS domain of SPI1, and is coexpressed with SPI1 in erythroid leukemia cells (Yamada et al., 2008). No other conserved binding sites for transcription factors know to be involved in B‐cell differentiation were identified. Therefore, we sought to test the hypothesis that SPI1 and HOXC13 may regulate *ZNF521/Zfp521* expression, either individually or synergistically.

### 2.3 SPI1 and HOXC13 regulate *Zfp521* expression

To determine if SPI1 and HOXC13 could act as *Zfp521* transcriptional regulators, we generated luciferase reporter constructs containing varying regions of the *Zfp521* promoter (Figure 2d). The 1Kb construct contains the HOXC13 and both SPI1 predicted binding sites, while the 0.5Kb promoter only contains the predicted SPI1 binding sites. The 0.2Kb promoter contains only the most proximal SPI1 binding site. We then tested the ability of SPI1 or HOXC13 to activate these various reporters in HEK293 cells. We found that when transfected individually, both SPI1 and HOXC13 proteins modestly activated the *Zfp521* luciferase reporter constructs. Upon cotransfection of SPI1 and HOXC13 we noted a greater than additive activation of the *Zfp521* reporter, confirming that SPI1 and HOXC13 activate *Zfp521* in a synergistic manner (Figure 2e). The greatest activation was detected from the 1Kb *Zfp521* promoter reporter construct containing all HOXC13 and SPI1 predicted binding sites (Figure 2e). Similar results were found when the reporter assays were performed in BCL1 B‐lymphoblast cells (data not shown).

### 2.4 SPI1 and HOXC13 regulation of *Zfp521* is dose‐dependent

**Figure 3:**
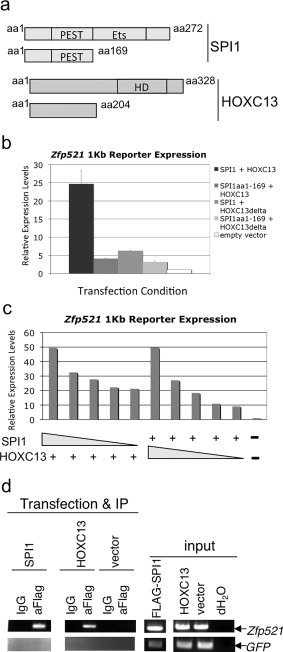
Analysis of *Zfp521* transcriptional activation by SPI1 and HOXC13. a: SPI1 and HOXC13 protein isoforms encoded by expression vectors. The full‐length isoforms are shown on top and truncation mutants shown below. Both truncation mutants remove the DNA binding domain from the protein. b: Reporter assay measuring *Zfp521* promoter activation following HEK293 cell cotransfection of wild type SPI1 and HOXC13 (black bar), transfection of truncated SPI1 and wild type HOXC13 (charcoal gray bar), transfection of wild type SPI1 and truncated HOXC13 (medium gray bar), or truncated SPI1 and truncated HOXC13 (light gray bar). Results were normalized to transfection with an empty expression vector (white bar). Only cotransfection of both wild‐type proteins demonstrated statistically significant gene activation when compared to empty expression vector control (t‐test; p < 0.05). Error bars represent standard deviation of 3 independent transfections, each with three technical replicates. c: SPI1 and HOXC13 regulation of the *Zfp521* promoter is dose‐dependent. Decreasing the amounts of transfection plasmid of either SPI1 or HOXC13 protein causes a decrease in promoter activation in HEK293 cells. The graph shows relative expression levels normalized to transfection with reporter construct only. d: Evaluation of SPI1 and HOXC13 binding to the *Zfp521* promoter. Transfection & IP: Protein‐DNA cross‐linking followed by immunoprecipitation of FLAG‐SPI1 or FLAG‐HOXC13 with an anti‐FLAG antibody allows amplification of the *Zfp521* promoter region, but immunoprecipitation using irrelevant antibody (IgG) does not precipitate the *Zfp521* promoter. Extracts from HEK293 cell transfections of the FLAG empty vector control do not show amplification of the *Zfp521* promoter following immunoprecipitation. The *GFP* vector control DNA is not detected in any samples following immunoprecipitation. Right panel: Input samples show the presence of *Zfp521* and *GFP* vector control DNA samples

We detected the greatest activation of *Zfp521* using the 1 Kb reporter construct, so we further examined *Zfp521* regulation using this construct for transfections in HEK293 cells. To confirm that the activation depended on SPI1 and HOXC13, we generated truncated versions of the SPI1 and HOXC13 proteins, lacking their respective DNA binding regions (Figure 3a). We confirmed that truncated mutant forms of SPI1 and HOXC13 proteins showed no *Zfp521* reporter activation above background levels, even when cotransfected with a wild‐type version of the partner protein (Figure 3b).

Following the observation that SPI1 and HOXC13 synergistically activate *Zfp521* expression, we examined the effects of varying the dosage of SPI1 and HOXC13 on *Zfp521* reporter activation. We performed cotransfection assays with decreasing amounts of either SPI1 or HOXC13 plasmid, while maintaining a constant concentration of the other construct. We found that the interaction between SPI1and HOXC13 is dose‐dependent (Figure 3c).

### 2.5 SPI1 and HOXC13 bind the *Zfp521* promoter

**Figure 4:**
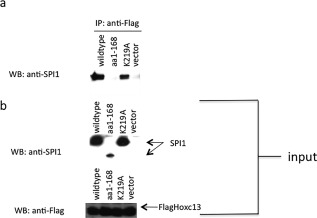
Protein‐protein interaction between Spi1 and HOXC13 tagged constructs. a: Co‐IP demonstrating that SPI1 and HOXC13 proteins have a physical interaction. The truncated form of SPI1 (aa1‐168) does not interact with HOXC13, but a missense mutant (K219A) of SPI1 does. b: Input samples. Mutant forms of SPI1 are shown (aa1‐168 and K219A). Vector = cotransfection with FLAG‐HOXC13 and empty vector lacking SPI1 sequence as negative control

A physical interaction between SPI1 and HOXC13 has been reported (Yamada et al., 2009). As our epitope‐tagged constructs varied slightly from the ones previously reported, we verified that our full‐length FLAG‐tagged HOXC13 protein did indeed bind to full‐length SPI1 (Figure 4). Following the confirmation that the SPI1 and HOXC13 proteins encoded by our epitope‐tagged constructs shared a physical interaction, we next examined whether they could also bind the *Zfp521* promoter directly. We performed chromatin immunoprecipitation assays on HEK293 cells transfected with either FLAG‐SPI1 or FLAG‐HOXC13 and the *Zfp521* 1Kb promoter construct. Following immunoprecipitation with an anti‐FLAG antibody, we detected an enrichment of the *Zfp521* promoter sequence from extracts individually transfected with either FLAG‐SPI1 or FLAG‐HOXC13 (Figure 3d). However, immuno‐precipitation with a nonspecific antibody (IgG) did not allow amplification of the *Zfp521* promoter. Likewise, transfection of the empty FLAG vector control did not produce enrichment of the *Zfp521* promoter following immunoprecipitation. As a control, we demonstrated that we could detect the *Zfp521* promoter in all input samples. We also detected a *GFP* vector control sequence in our input samples, but not in any reactions subjected to immunoprecipitation. The enrichment of the *Zfp521* promoter region following immunoprecipitation of either SPI1 or HOXC13 indicates that SPI1 and HOXC13 proteins bind the *Zfp521* promoter (Figure 3d) in a specific manner.

**Figure 5:**
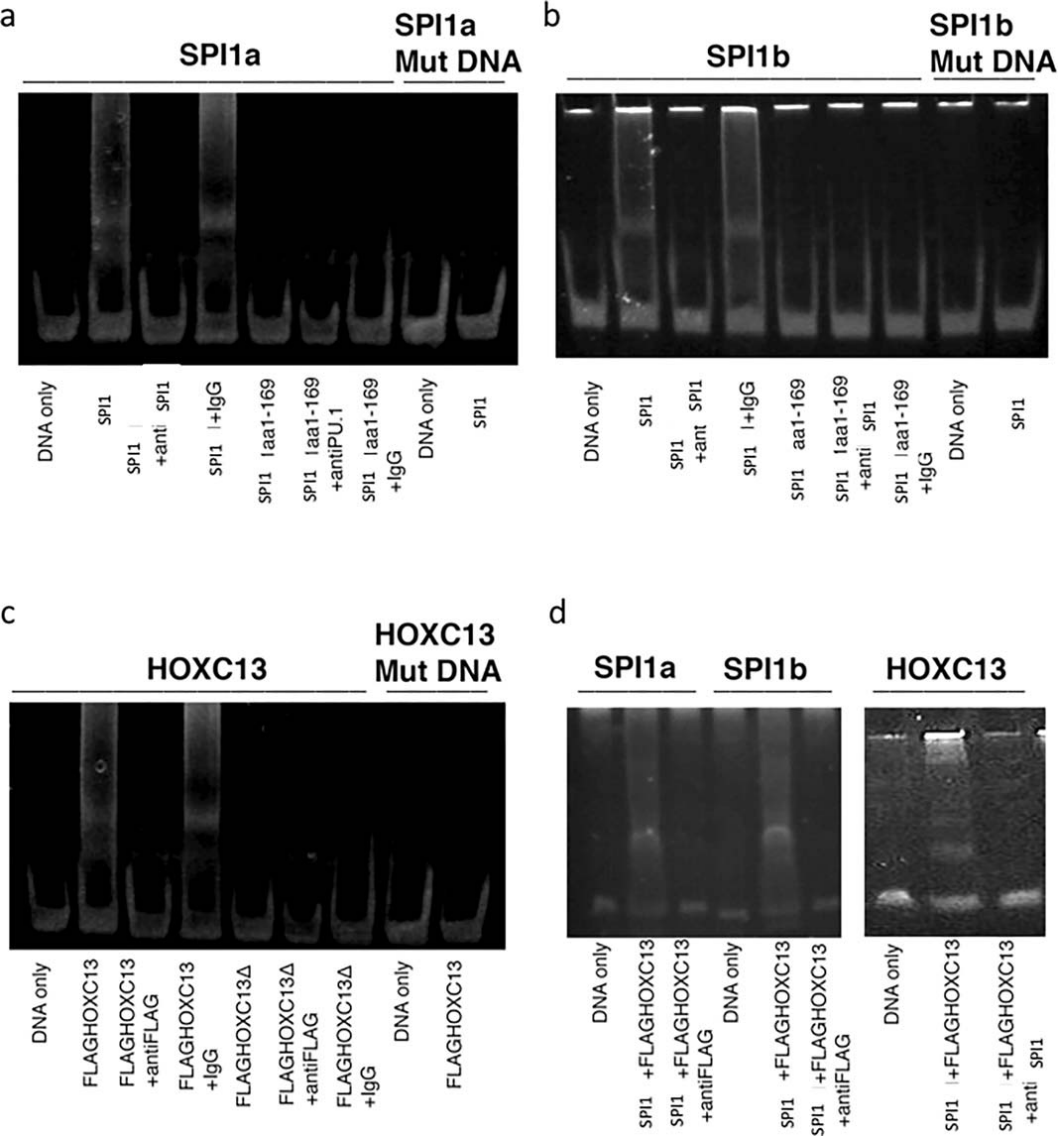
*In vitro* assessment of SPI1 and HOXC13 binding to the *Zfp521* promoter predicted binding sites. DNA sequences added to assay are shown above gels, and protein extracts added are shown below gels for each lane. a: EMSA reactions demonstrate that full‐length SPI1 binds to the predicted SPI1a binding site of the *Zfp521* promoter, because the migration of the DNA probe is reduced when protein extract is added prior to electrophoresis (arrow). Addition of an anti‐SPI1 antibody abrogates the shift seen in DNA migration, while addition of a nonspecific antibody (IgG) does not affect the shift in DNA migration. The truncation mutant form of SPI1 (aa1‐169) is not capable of binding to the *Zfp521* promoter sequence. Adding specific or nonspecific antibody with the truncation mutant does not affect migration of the *Zfp521* promoter SPI1a DNA sequence. A mutated version of the *Zfp521* promoter SPI1a site is not bound by wild type SPI1 protein. b: EMSA reactions demonstrate that full‐length SPI1 binds to the predicted SPI1b binding site of the *Zfp521* promoter (arrow). Addition of an anti‐SPI1 antibody abrogates the shift seen in DNA migration, while addition of a nonspecific antibody (IgG) does not affect the shift in DNA migration. The truncation mutant form of SPI1 (aa1‐169) is not capable of binding to the *Zfp521* promoter sequence. Adding specific or nonspecific antibody with the truncation mutant does not affect migration of the *Zfp521* promoter SPI1b DNA sequence. A mutated version of the *Zfp521* promoter SPI1b site is not bound by wild type SPI1 protein. c: EMSA reactions demonstrate that full‐length FLAG‐HOXC13 binds to the predicted Hoxc13 binding site of the *Zfp521* promoter (arrow). Addition of an anti‐FLAG antibody abrogates the shift seen in DNA migration, while addition of a nonspecific antibody (IgG) does not affect the shift in DNA migration. The truncation mutant form of Hoxc13 (Hoxc13delta) is not capable of binding to the *Zfp521* promoter sequence. Adding specific or nonspecific antibody with the truncation mutant does not affect migration of the *Zfp521* promoter Hoxc13 DNA sequence. A mutated version of the *Zfp521* promoter Hoxc13 site is not bound by wild type FLAG‐Hoxc13 protein. d: The use of the anti‐FLAG antibody (recognising FLAG‐HOXC13) eliminates the shift of the *Zfp521* promoter predicted SPI1a and SPI1b binding sites (arrow), similar to the results seen for SPI1 protein and anti‐SPI1 antibody on its own predicted promoter site (Figure 5a‐b). The use of the SPI1 specific antibody eliminates the shift of the *Zfp521* promoter predicted HOXC13 binding site, similar to the results seen for HOXC13 protein and anti‐FLAG antibody on its own predicted promoter site (Figure 5c). As shown in Figure 2c, the SPI1 binding site further away from the *Zfp521* transcriptional start site was called SPI1a, and the SPI1 site closer to the *Zfp521* transcription start site termed SPI1b

To provide further support for the hypothesis that SPI1 and HOXC13 bind the *Zfp521* promoter, we assayed binding *in vitro* via EMSAs. We confirmed that the full‐length SPI1 and HOXC13 proteins were indeed capable of binding to their predicted recognition sequences in the *Zfp521* promoters, as indicated by reduced migration of the promoter DNA (Figure 5a–c). SPI1 and HOXC13 truncation mutants did not alter DNA migration. The addition of specific antibodies (anti‐SPI1 or anti‐FLAG) abolished the band shift observed in the EMSA, suggesting that the binding of the antibody interfered with the DNA‐binding activity of the SPI1 and FLAG‐HOXC13 proteins. The addition of a nonspecific antibody (IgG) did not disrupt the migration of the promoter DNA sequence. Additionally, incubating the nonspecific antibody with the SPI1 and HOXC13 truncation mutant proteins did not produce a shift in DNA migration, demonstrating that the binding seen in reactions with the nonspecific antibody is due to SPI1 and HOXC13 proteins rather than the antibody.

We also wished to examine whether SPI1 and HOXC13 were colocalized on the *Zfp521* promoter, in support of our finding that these proteins synergistically activate *Zfp521* expression. We therefore sought to determine if the presence of anti‐FLAG antibody (detecting FLAG‐HOXC13 transfected protein) in a binding reaction including both SPI1 and HOXC13 full‐length proteins would disrupt band shifting of either of the SPI1 binding sites of the *Zfp521* promoter. A reciprocal reaction was performed using the SPI1 antibody and the putative HOXC13 binding site of the *Zfp521* promoter. In all cases we found that the presence of the partner antibody disrupted the band‐shift (Figure 5d) in a manner similar to that seen for the specific antibodies (Figure 5a–c). From these results we conclude that the SPI1 and HOXC13 proteins are closely associated at their predicted binding site sequences in the *Zfp521* promoter, supporting the model for synergistic regulation of *Zfp521* expression.

### 2.6 Knockdown of *SPI1* and *HOXC13* reduces *ZNF521* expression

**Figure 6:**
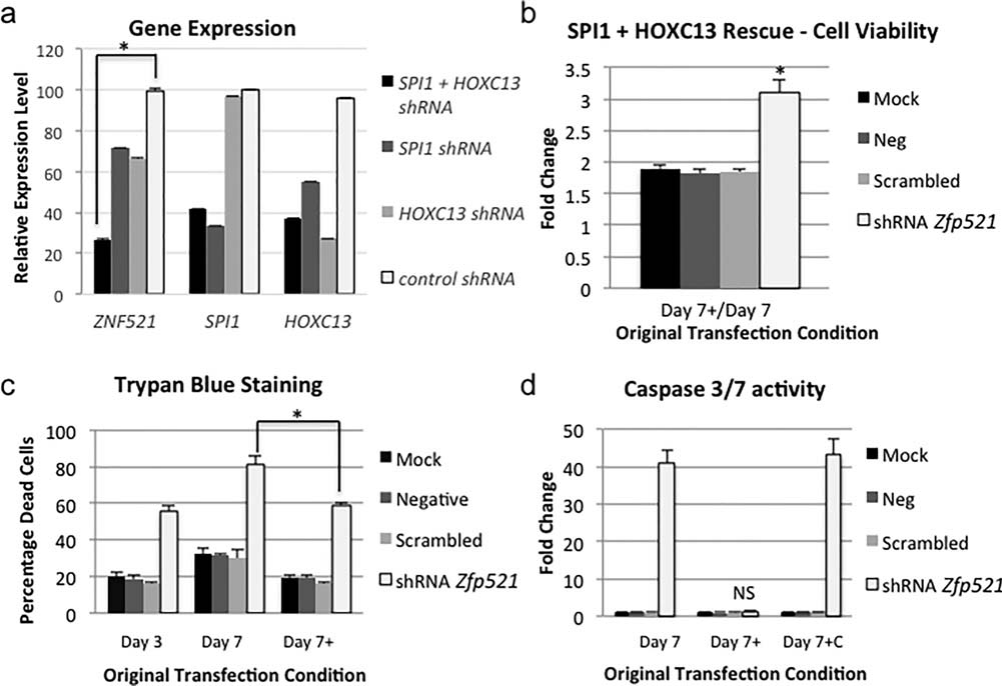
Knockdown of *SPI1* and *HOXC13* reduces *ZNF521* expression, and expression of *Spi1* and *HOXC13* can rescue *Zfp521* knockdown cell defects. a: *SPI1* and *HOXC13* were knocked down by shRNA transfection in THP‐1 cells, either in combination (black bars) or individually (gray bars). A control vector with a scrambled noneffective shRNA sequence was used as a control (light gray bars). Gene expression of *ZNF521*, *SPI1*, and *HOXC13* were assayed in each knockdown condition (labels on x‐axis). Knockdown of both *SPI1* and *HOXC13* resulted in significantly reduced *ZNF521* expression as compared to control shRNA transfection (t‐test; p < 0.05). Individual knockdowns had a less profound reduction in *ZNF521* expression levels. Expression levels of *SPI1* and *HOXC13* confirm that the shRNA transfections reduced gene expression of each gene as expected. b: Using a knockdown rescue assay, we compared the ratio of viable cells on day 7 post‐transfection for cells that were initially transfected with *Zfp521* knockdown shRNA plasmids or control plasmids to cells that had the original knockdown plus a transfection on day 3 of *Spi1* and *HOXC13* expression constructs (day 7+). Cell viability is recovered when *Spi1* and *HOXC13* are cotransfected into BCL1 cells after *Zfp521* shRNA transfection, and shows a significant increase when compared to cells with initial mock transfection (t‐test; p < 0.05). Cells with initial empty vector or a scrambled *Zfp521* shRNA sequence transfection do not show a similar increase in cell viability after rescue transfection. c: The percentage of dead cells identified by trypan blue staining in BCL1 cell cultures following *Zfp521* knockdown or control transfections on day 3 (left) is lower than the percentage of cells on day 7 (middle). When *Spi1* and *HOXC13* expression constructs are cotransfected on day 3, the percentage of dead cells on day 7+ (right) is reduced as compared to cells on day 7 that did not receive rescue plasmid transfections (t‐test, p < 0.05). d: Caspase 3/7 activity was measured in BCL1 cells 7 days after transfection with *Zfp521* shRNA or control plasmids (left). Introduction of *Spi1* and *HOXC13* expression vectors by cotransfection on day 3 resulted in a significant reduction in Caspase 3/7 activity on day 7+ (middle), with no significant difference in cells initially transfected with *Zfp521* shRNA as compared to cells with any other initial transfection condition. Cells transfected with a control empty vector on day 3 did not show a similar reduction in Caspase 3/7 activity (day 7 + C; right). NS = nonsignificant. In all panels error bars represent standard deviation of 3 independent transfections, each with three technical replicates

To confirm that SPI1 and HOXC13 are required for activation of *Zfp521*, we performed a knockdown experiment. shRNA plasmids containing sequences targeting *SPI1* and *HOXC13* were transfected either individually or jointly into THP‐1 cells. A plasmid with a scrambled shRNA sequence was used as a control. Expression levels of *SPI1, HOXC13*, and *ZNF521* were measured in each transfection condition by qPCR. We found that *ZNF521* expression levels were significantly reduced upon cotransfection of the *SPI1* and *HOXC13* shRNA constructs (Figure 6a) as compared to the scrambled shRNA control. Transfection of either the *SPI1* or *HOXC13* shRNA constructs individually reduced *ZNF521* expression, but the reduction was not as great as the cotransfection condition. *SPI1* expression and *HOXC13* expression were each reduced following the transfection of their specific shRNA construct, but not by transfection with the control shRNA plasmid (Figure 6a).

### 2.7 *SPI1* and *HOXC13* expression rescues the effects of *Zfp521* knockdown

Using the mouse BCL1 B‐lymphoblast cell line, we have demonstrated that knockdown of *Zfp521* reduces cell viability, increases apoptosis, and alters expression of Pro‐B‐cell genes (Al Dallal et al., 2016). To provide further evidence for a role for SPI1 and HOXC13 in *Zfp521* regulation, we also assessed whether addition of these factors could restore cell viability following knockdown of *Zfp521* in a BCL1 cells. We therefore cotransfected SPI1 and HOXC13 into BCL1 cells 3 days after an initial transfection with the *Zfp521* shRNA or experimental control plasmids. On day 7 after the initial transfection, the ratio of viable cell number from cells receiving the rescue plasmids (denoted 7+) compared to control cells receiving no rescue plasmid (denoted 7) was calculated. We found that cotransfection of SPI1 and HOXC13 into cells with *Zfp521* knockdown led to a significant increase in viable cell number, as compared to cells with control transfections (Figure 6b). The percentage of dead cells, as indicated by trypan blue staining, was reduced in *Zfp521* knockdown cells subsequently cotransfected with SPI1 and HOXC13 as compared to cells with a mock transfection (Figure 6c). Additionally, levels of activated CASPASE 3/7, indicative of apoptosis, were significantly reduced in *Zfp521* knockdown cells cotransfected with SPI1 and HOXC13, as compared to cells with mock transfection (Figure 6d). A rescue transfection performed with an irrelevant empty vector (pCDNA3.1, denoted day 7 + C) did not show a reduction in activated caspase 3/7 activity (Figure 6d). Because trypan blue stains both necrotic and apoptotic cells, we attribute the presence of trypan blue positive cells in the SPI1 and HOXC13 cotransfection condition (Figure 6c) to necrosis, since caspase 3/7 staining reveals minimal apoptosis in this sample (Figure 6d).

### 2.8 *Hoxc13*, *Spi1*, and *Zfp521* are coexpressed in immune tissues

**Figure 7:**
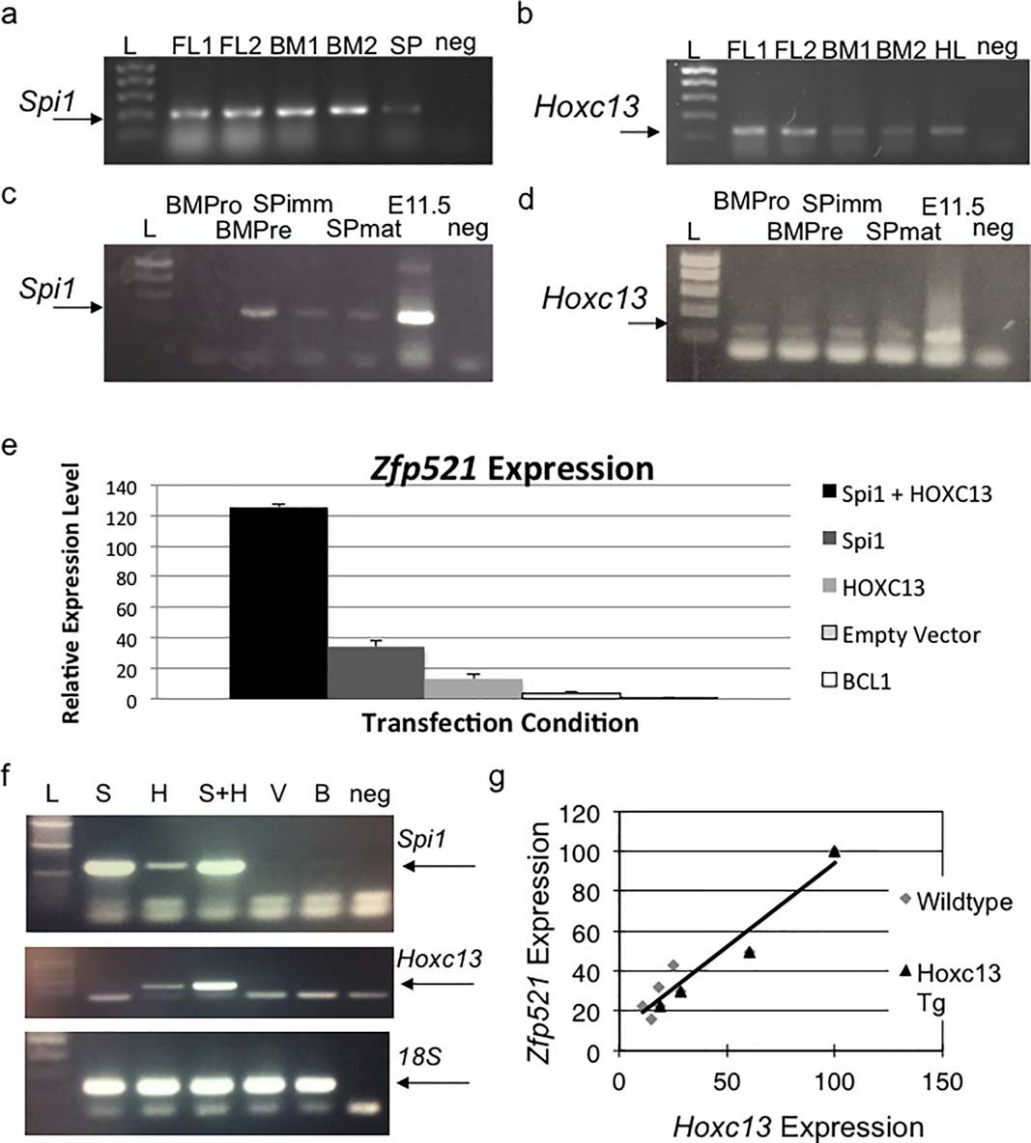
Assessment of *Spi1*, *Hoxc13*, and *Zfp521* expression in immune tissues. a: The expression of *Spi1* as detected by RT‐PCR in mouse fetal liver (FL) and adult bone marrow (BM) samples. Spleen cDNA (SP) is used as a positive control, and no template as a negative control (neg). Bioline Hyperladder I (L) is used as a molecular weight standard. b: The expression of *Hoxc13* as detected by RT‐PCR in mouse immune tissues (samples as in A). Hindlimb (HL) is used as a positive control. c: Expression of *Spi1* in FACS sorted B‐cells. Expression was analysed in ProB cells isolated from bone marrow (BMPro), PreB cells isolated from bone marrow (BMPre), immature B‐cells isolated from spleen (SPimm), and mature B‐cells isolated from spleen (SPmat). Embryonic day 11.5 (E11.5) cDNA was used as a positive control. d: Expression of *Hoxc13* in cDNA samples (samples as in C). e: Quantitative RT‐PCR demonstrates that cotransfection of SPI1 and HOXC13 into mouse BCL1 cells results in up‐regulation of endogenous *Zfp521* expression (black bar). The up‐regulation is greater than for cells transfected with either protein individually, cells with no transfection, or empty expression vector. Results show expression normalized to *18S* levels, reported relative to BCL1 mock transfected cells (BCL1). Error bars show standard deviation of three independent experiments, each with three technical replicates. f: RT‐PCR on BCL1 cells used for quantitative expression analysis confirms expression of *Spi1* and *HOXC13* from transfection plasmids. g: The expression levels of *HoxC13* and *Zfp521* were compared to each other within fetal liver samples from the same embryo. A correlation is seen between the relative expression levels of *Hoxc13* and *Zfp521* in mouse fetal liver from GC13 transgenic (Tg) mice (black triangles) and nontransgenic (wildtype) littermate controls (gray diamonds; *R* ^2^ = 0.91). Expression was measured by quantitative RT‐PCR and normalized to *Gapdh*

During embryonic development B‐cells begin to differentiate in the fetal liver (Yokota et al., 2006). If SPI1 and HOXC13 cooperatively regulate *Zfp521* expression during B‐cell differentiation, we hypothesized that they should both be expressed in the fetal liver. We found that both genes are expressed in mouse fetal liver from two separate animals at E16.5. Expression is also detected in adult mouse bone marrow, a well‐documented location for B‐cells at all stages of differentiation (Figure 7a,b). To better define *Spi1* and *Hoxc13* expression during B‐cell differentiation, we FACS sorted B‐cells from mouse bone marrow and spleen into different subpopulations. *Zfp521* expression in these sub‐populations has been confirmed (Hiratsuka et al., 2015). *Spi1* expression was not detected in bone‐marrow derived pro‐B‐cells, but was present in bone marrow derived pre‐B‐cells, spleen derived immature B‐cells, and spleen derived mature B‐cells (Figure 7c). We found that *Hoxc13* was expressed in all subpopulations examined (Figure 7d). These data support the hypothesis that SPI1 and HOXC13 are developmental regulators of *Zfp521* expression.

### 2.9 SPI1 and HOXC13 cooperatively up‐regulate *Zfp521* expression in vivo

We cotransfected *Spi1* and *HOXC13* expression vectors into the mouse B‐lymphoblast BCL1 cell line, and tracked endogenous *Zfp521* expression levels through qPCR. We found that endogenous *Zfp521* expression increased with cotransfection of wild‐type SPI1 and HOXC13 (Figure 7e). However, *Zfp521* expression levels were not as greatly increased following either SPI1 or HOXC13 single transfections. This result indicates that SPI1 and HOXC13 do synergistically regulate *Zfp521* expression in B‐cells. An analysis of the same BCL1 cell cDNA samples by RT‐PCR confirms the expression of *Spi1* and *Hoxc13* from transfection plasmids in these samples (Figure 7f). Similar results were obtained for transfections performed in Ba/F3 cells (Supporting Information Figure 1).

To extend our analysis, we also examined fetal liver expression of *Zfp521* in embryos of Tg(Hoxc13)^61B1Awg^ mice, a transgenic strain over‐expressing *Hoxc13* (Tkatchenko et al., 2001). The relative expression levels of both genes were compared within the same mouse embryo. Transgenic embryos with high levels of *Hoxc13* expression showed a significant increase in *Zfp521* expression in the liver at E16.5 as compared to nontransgenic littermates (Figure 7g). Notably, not all Tg(Hoxc13)^61B1Awg^ embryos demonstrated increased *Hoxc13* expression in the liver, perhaps due to epigenetic alterations of transgene expression, but the expression levels of *Hoxc13* and *Zfp521* were strongly correlated in all embryos (R^2^ = 0.91). As the fetal liver is a critical developmental location of B‐cell differentiation (Yokota et al., 2006), we conclude that the HOXC13‐dependent mechanism of *Zfp521* regulation is relevant *in vivo*.

## 3 Discussion

The large, multi‐zinc finger proteins ZFP521 and ZFP423 were initially characterized as proto‐oncogenes, both causing B‐cell leukemia in mouse models (Hentges et al., 2005; Warming et al., 2003; Warming et al., 2004). Subsequently the critical roles that these factors play in cellular differentiation have been identified, and on‐going investigations will likely reveal additional details about the mechanisms by which they regulate gene expression. Both proteins contain zinc‐fingers of the Krüppel type, which are predicted to meditate protein‐protein interactions as well as possess DNA‐binding ability. Whilst protein binding partners for ZFP521 have been identified, such as EBF (Mega et al., 2011), the DNA sequence to which ZFP521 binds is as yet unknown. Likewise, the DNA sequence bound by ZFP423 has not been identified. Although both proteins have been shown to regulate cellular differentiation processes, the overlap between their functions at a molecular level, such as whether they will both bind the same DNA sequence, is unclear. Our phylogenetic data combined with prior reports of different expression patterns for these two genes suggests that their expression patterns have diversified following a gene duplication event, which may explain why both genes were retained in vertebrate genomes.

As *Zfp521* expression is detected early in the B‐cell differentiation process (Hiratsuka et al., 2015; Warming et al., 2003), relevant transcription factors that initiate *Zfp521* expression should be expressed at early stages of B‐cell differentiation. In fact, SPI1 expression is found in committed lymphoid progenitor cells (DeKoter et al., 2002), making SPI1 a good candidate for the regulation of *Zfp521* during B‐cell differentiation. *Spi1*, *Hoxc13*, and *Zfp521* are all expressed in the fetal liver, where B‐cell differentiation originates during development (Yokota et al., 2006). Importantly, we find that if we cotransfect SPI1 and HOXC13 into mouse BCL1 cells, a B‐lymphoblast cell line, the endogenous expression levels of *Zfp521* in the cells are up‐regulated (Figure 4e). *Hoxc13* over‐expressing mice display up‐regulation of *Zfp521* in the fetal liver, providing further supporting evidence that SPI1 and HOXC13 coregulation of *Zfp521* expression is relevant during developmental specification of B‐lymphocytes.

*Zfp521* up‐regulation resulting from retroviral insertions at the 5' end of the *Zfp521* locus causes B‐cell transformation in mice (Hentges et al., 2005; Warming et al., 2003). The tumors present in these mice express a variety of cellular markers known to be downstream of the B‐cell transcription factor EBF, which are indicative of activated B‐cell receptor (BCR) signalling (Hentges et al., 2005). Additionally, retroviral insertion up‐regulation of *Zfp521* has been identified as an important cooperative event which increased the incidence of B‐ALL in mice expressing an *E2A‐HLF* transgene as a model for human t(17;19) acute lymphoblastic leukemia (Yamasaki et al., 2010). The finding that SPI1 and HOXC13 coregulate *Zfp521* expression highlights the possibility that over‐expression of either of these factors could lead to B‐cell leukemia via activation of *Zfp521*, resulting in enhanced BCR signalling in a manner similar to that displayed in AKXD27 mice (Hentges et al., 2005). During the process of bone differentiation *Cyclin D1* has been identified as a ZFP521 target gene, promoting proliferation of growth plate chondrocytes (Correa et al., 2010). Tumors from mice with retroviral insertions within the *Zfp521* gene have increased *Cyclin D1* expression (Al Dallal et al., 2016), suggesting that over‐expression of *Zfp521* leads to an excessive proliferation of immature B‐lymphocytes via *Cyclin D1* up‐regulation, culminating in B‐cell leukemia. In humans *ZNF521* over‐expression is found in acute myeloid leukemia but not in B‐cell leukemia (Mesuraca et al., 2015), although B‐cell leukemia initiating cells do show increased expression of *ZNF521* (Aoki et al., 2015). It has thus been proposed that up‐regulation of *ZNF521* in rare leukemia initiating cells may facilitate tumor progression, even though *ZNF521* over‐expression is not detected in the majority of leukaemic B‐cells from patients (Mesuraca et al., 2015).

The role of SPI1 in leukemia is complex. Over‐expression of SPI1 is known to cause erythro leukemia by inhibiting apoptosis and blocking the terminal differentiation of erythrocytes (Yamada et al., 2008). STAT3 activation of SPI1 is a key step in the disease progression of Friend erythro leukemia (Hegde et al., 2009), reinforcing the finding that SPI1 over‐expression alters erythroid cell differentiation. However, acute myeloid leukemia can result from loss‐of‐function mutations in *SPI1* or reduced Spi1 expression (Sive et al., 2016; Will et al., 2015), while induced expression of SPI1 in myeloid leukemia cells restores their differentiation and reduces their proliferation rates (Tkatchenko et al., 2001). Likewise, expression of SPI1 is severely reduced in human patients with chronic myeloid leukemia due to aberrant promoter methylation in tumor cells (Yang et al., 2012). Deletion of SPI1 and the related ETS‐transcription factor SPI‐B in the B‐cell lineage results in fully‐penetrant pre‐B‐cell acute lymphoblastic leukemia (B‐ALL), suggesting that SPI1 and SPI‐B are tumor suppressors in the B‐cell lineage (Sokalski et al., 2011). Likewise, mice lacking both SPI1 and IRF8 develop B‐ALL (Pang et al., 2016). These results suggest that over‐expression of SPI1 may play a part in erythroid leukemia, while reduced SPI1 expression contributes to myeloid and B‐cell leukemia, although the many diverse functions of SPI1 at multiple time points during hematopoiesis complicate the analysis of SPI1 in specific lineages.

HOXC13 is a key downstream target of the polycomb group family gene B‐cell specific Moloney murine leukemia virus integration site 1 (BMI‐1). BMI‐1 dysregulation leads to lymphoma and nonsmall cell lung carcinoma in humans (Jacobs et al., 1999; Vonlanthen et al., 2001). Additionally, in human cervical adenocarcinoma cells *BMI‐1* knockdown promotes the up‐regulation of *HOXC13* expression, contributing to a block in cell proliferation (Chen et al., 2011). Therefore, BMI‐1 induced *HOXC13* repression may cause abnormal cell proliferation contributing to adenocarcinoma. HOXC13 has additional links to leukemia, because fusions of NUP98 and HOXC13 cause human myeloid leukemia (Cheng & Reed, 2007). Also, an antagonistic role for SPI1 and HOXC13 in erythroid cell differentiation has been proposed based on data from erythro leukemia cell lines (Yamada et al., 2008). Cross‐talk between SPI1 and the MEIS/HOX gene regulation pathway has been noted in mixed lineage leukemia (Zhou et al., 2014). Yet to our knowledge there are no prior reports of HOXC13 involvement in lymphoid leukemia. However, based on the results of this study, the potential role of HOXC13 in lymphoid leukemia and B‐cell gene expression via regulation of *Zfp521* should be explored. Future experiments are required to determine the contribution of perturbations in *Hoxc13* and *Spi1* expression to dysregulation of *Zfp521*, and the potential for alternations in the expression of these transcription factors to promote lymphoid leukemia.

## 4 Materials and methods

### 4.1 Phylogenetic analysis

Protein sequences for orthologues of both ZFP521 and ZFP423 from multiple species were obtained from the online database of National Centre for Biotechnology Information (Supporting Information Table 1). Multiple sequence alignment was performed using MUltiple Sequence Comparison by Log‐Expectation (MUSCLE) (Edgar, 2004). Phylogenetic trees were generated by MUSCLE, and branch length presented as either cladogram (Figure 1a) or real (Figure 1b).

### 4.2 Bioinformatic promoter analysis

The 1Kb regions upstream of the annotated mouse and human *Zfp521/ZNF521* and *Zfp423/ZNF423* genes were obtained from the UCSC genome browser using the mm9 assembly for mouse and the hg38 assembly for human. Annotated tracks for DNAse I hypersensitivity (Ho & Crabtree, 2010) and the percentage of GC bases in the promoter regions were obtained from the UCSC genome browser on the hg38 and mm9 assemblies. DNase I hypersensitivity data was generated by the University of Washington ENCODE group (Sabo et al., 2006). The 1Kb mouse and human promoter regions were searched for transcription factor binding sites using TESS (Schug, 2008). Conserved sites were identified through manual inspection of annotated promoters.

### 4.3 Cell culture

The BA/F3 cell line was maintained in RMPI with 2% mouse interleukin‐3, 10% FBS in 5% CO_2_ at 37°C. BCL1 mouse lymphoblast cells (ATCC® TIB‐197) were cultured in RPMI 1640 medium supplemented with 2mM L‐glutamine (Lonza), 0.05mM 2‐mercaptoethanol (Sigma‐Aldrich), 15% Foetal Bovine Serum (FBS), 5% penicillin, 5% streptomycin at 37°C with 5% CO2. THP‐1 cells were cultured in RMPI with 0.05 mM β‐Mercaptoethanol and 10% FBS. The HEK293 cell line was maintained in DMEM with 10% FBS in 5% CO_2_ at 37°C.

### 4.4 Constructs

IMAGE clone 3600260 was used as the wild‐type *Spi1* expression construct and as the template for site‐directed mutagenesis. IMAGE clone 6171228 was used as the wild‐type *HOXC13* expression vector. Primer sequences for *Zfp521* promoter amplification listed in Supporting Information Table 2. Promoter regions were cloned up stream of the luciferase gene in the pGL3 Basic plasmid. Nomenclature of the reporter plasmids is based on the approximate size of promoter regions. The human *HOXC13* cDNA sequence was cloned in‐frame into pFLAG‐CMV2 (Sigma) to create the FLAG‐tagged expression construct. The HOXC13 DNA‐binding mutant construct (HOXC13‐delta) was previously described (Potter et al., 2006).

### 4.5 Site‐directed mutagenesis

Site‐directed mutagenesis was performed using Pfu Turbo DNA polymerase (Stratagene). Cycling conditions: 95.0°C for 30 seconds; followed by 12 cycles of: 95.0°C for 30 seconds, 55.0°C for 1.00 minute, 72.0°C for 6.00 min, and final hold at 37.0°C. PCR products were subjected to *DpnI* (NEB) digestion at 37.0°C for 4 h. Digested DNA was transformed and sequenced to confirm the presence of the point mutation creating a stop codon at SPI1 amino acid 170. Primer sequences are listed in Supporting Information Table 2.

### 4.6 Transfection assays

0.5µg plasmid DNA was transfected into 5 × 10^5^ HEK 293 cells with Fugene 6 (Roche), and cells were cultured for 48 h. 2 μg plasmid was transfected into 2 × 10^6^ BA/F3 cells with Amaxa nucleofector reagent (Lonza). Cells were grown for 48 h to collect total RNA or protein for analysis. 1 µg plasmid DNA was transfected into 1 x 10^5^ BCL1 cells with FuGENE HD (Roche) in OptiMEM Media (Sigma). Cells were grown for 48 h for qPCR assays, and 3 or 7 days for knockdown rescue assays.

### 4.7 Western blot

Cell lysate was prepared in RIPA buffer. 50 μg total protein was subjected to 12% SDS‐PAGE and transferred to PVDF membrane. The membrane was incubated with mouse anti‐FLAG antibody (M2, Sigma) or goat anti‐SPI1 antibody (anti‐PU.1 D19, Santa Cruz). Protein was detected by ECL kit (GE Health care).

### 4.8 Luciferase assay

0.1μg pSPORT6‐Spi1 and 0.1 μg pSPORT6‐HOXC13 were cotransfected into 1.25 × 10^5^ HEK293 cells with 0.05 μg pGL3‐basic vector containing 0.2Kb, 0.5Kb or 1Kb *Zfp521* promoter regions and 0.001ug pRLCMV using Amaxa nucleofector reagent (Lonza). Luciferase activity was determined by dual‐luciferase Reporter Assay System (Promega). Ratios of firefly luciferase activity to renilla luciferase activity were calculated for each sample. Reactions were performed in triplicate, and all results represent combined analysis of three separate experiments. Expression levels are shown relative to transfection of luciferase reporter without cotransfection of protein expression constructs. Statistical significance was determined by t‐test comparison to reporter control.

### 4.9 Protein coimmunoprecipitation

HEK 293 cells cotransfected with 2 μg of SPI1 (wild type or mutant constructs) and Flag‐HOXC13 were harvested 48 h following transfection and lysed in PBS with 1% Triton X100 and 0.01% Igepal CA‐630. The lysate was incubated with 2 μg anti‐Flag (Sigma) or anti‐SPI1 antibody (anti‐PU.1 D19, Santa Cruz) and 20 μl protein G Dynabeads (Invitrogen) overnight, then washed with PBS with 1% Triton X100 and 0.01% Igepal CA‐630 and subjected to Western blot.

### 4.10 Chromatin immunoprecipitation

HEK 293 cells were transfected with pFlagCMV‐Spi1 or pFlagCMV‐HOXC13. Cells were also transfected with the pGL3‐1kb *Zfp521* promoter or pGL3‐GFP plasmid control vector. The day before cells were harvested, 5 μl protein G Dynabeads were pre‐incubated with 0.1mg/ml salmon sperm DNA and 1mg/ml BSA. 24 hours after transfection, the cells were fixed by 4% formaldehyde for 5 minutes, and the reaction was stopped by the addition of 0.125M glycine. Cells were washed twice with PBS and lysed with lysis buffer (0.1M NaCl, 1mM EDTA, 1% Triton‐X 100, 50 mM Tris‐HCl pH7.5). After the lysate was pre‐cleared with pre‐blocked Dynabeads, 1 μg anti‐Flag antibody was added to the lysate, and incubated at 4°C overnight. The lysate was incubated with 5 μl Dynabeads for 1 hour, and then washed with low salt buffer (1% Triton X‐100, 2 mM EDTA, 20 mM Tris‐HCl pH8.1, 150 mM NaCl), high salt buffer (1% Triton X‐100, 2 mM EDTA, 20 mM Tris‐HCl pH8.1, 500 mM NaCl), LiCl buffer (0.25 M LiCl, 1% Igepal CA‐630, 1 mM EDTA, 10 mM Tris‐HCl pH8.1) and TE buffer. After washing, beads were incubated in elution buffer (1%SDS, 0.1M NaHCO3) twice, each 10 minutes. Elution buffer was collected and adjusted to a final concentration of 0.2 M NaCl, and then incubated at 65°C overnight. The eluate was RNase digested for 1 hour, incubated in proteinase K for 2 hours and purified by Qiaquick PCR purification kit (Qiagen). DNA was amplified with Faststart Taq DNA polymerase (Roche) with GC RICH solution with *Zfp521* promoter primers or *GFP* plasmid primers listed in Supporting Information Table 2. PCR conditions were 95°C 4 minutes, and then followed by 95°C 30 s, 60°C 30 s and 72°C 1 min for 35 cycles, and 72°C 10minutes.

### 4.11 Electrophoretic mobility shift assay (EMSA)

Following transfection with pCMVSPORT6‐Spi1 and pCMV2FLAG‐HOXC13, BCL1 adherent cells were trypsinized into 2 ml Trypsin (Gibco), pelleted by centrifugation, and the cell pellet re‐suspended into cold high salt buffer (RIPA). Cells were lysed on ice for 15 minutes and then centrifuged for 30 minutes at 4°C. The supernatant was collected for use in binding reactions. Prior to use in EMSA, DNA oligonucleotides were denatured at 95°C for 5 minutes, and left to cool to room temperature. DNA probe sequences are listed in Supporting Information Table 2. Extracts were incubated with double stranded DNA fragments containing predicted SPI1 and HOXC13 binding sites of the *Zfp521* promoter and EMSA binding buffer (10mM Tris‐HCL pH 7.4, 150mM KCL, 0.1mM EDTA and 0.1mM DTT) at room temperature for 20 minutes, according to manufacturer's instructions (Life Technologies SYBR Green EMSA kit cat#E‐33075). For reactions containing antibodies, anti‐SPI1 (anti‐PU.1 D‐19, Santa Cruz), anti‐FLAG (M2, Sigma), or anti‐IgG (Santa Cruz) antibody was added to the reaction mix prior to room temperature incubation.

The products were resolved by electrophoresis on 6% nondenaturing polyacrylamide gel at 150 V in 1.0 X TBE buffer for 45 minutes. DNA migration was determined by staining the gel with SYBR Green (1:10,000; Molecular Probes, Eugene, OR) for 20 min at room temperature, and rinsing twice with deionized H_2_O. The gel was visualized with a Dark Reader Transilluminator.

### 4.12 *SPI1* and *HOXC13* knockdown assay

0.25ug shRNA targeting *SPI1* (Origene TL316738) and/or shRNA targeting *HOXC13* (Origene TL304059) were transfected into 2X10^6^ THP‐1 cell with Amaxa nucleofector (Lonza VCA‐1003); a scrambled noneffective shRNA sequence was used as a negative control. After 3 days, RNA was isolated from transfected cells and cDNA was prepared. The expression level of *ZNF521, SPI1* and *HOXC13* was determined by qPCR as described below for mouse cell line and tissue samples. The qPCR primers are listed in Supporting Information Table 2.

### 4.13 *Zfp521* shRNA knockdown rescue assays

Knockdown of *Zfp521* was performed by transfection of a cocktail of 4 vectors with shRNAs targeting *Zfp521* into BCL1 cells as previously described (Al Dallal et al., 2016). The rescue assay with SPI1 and HOXC13 was performed by cotransfecting 0.5 μg each of *Spi1* and *HOXC13* expression constructs into knockdown or control cells 3 days after *Zfp521* shRNA transfection. As a control, a rescue transfection was performed with pcDNA3.1 empty vector on day 3 post‐shRNA transfection. Cell viability, trypan blue staining, and Caspase 3/7 activity analysis were performed as previously described (Al Dallal et al., 2016).

### 4.14 Flow cytometry

Bone marrow was extracted from the femurs of two 129S5 mice by flushing the bones with DMEM, pooling the cells, passing the cells through a 21 gauge needle and filtering the cells using a 0.7um filter (BD). Splenocytes were isolated by pressing the spleen from 2 mice against a mesh, then suspending the cells in DMEM. Erythrocytes were lysed by incubating the cells with red blood cell lysis buffer (Roche) for 5 minutes at room temperature. Cells were washed with FACs buffer (PBS, 1% FCS, 0.05% sodium azide), pelleted and resuspended with 1ug of FC receptor block and incubated for 20 minutes at 4°C. The cells were washed in FACs buffer, and resuspended in 0.313ug/ml anti‐B220 (RA3‐6B2) APC (ebioscience), 2.5ug/ml anti‐IgM (Il/41) PE (ebioscience), 0.625ug/ml anti‐IgD (11‐26c) Fitc (ebioscience), 2.5 μg/ml anti‐C‐kit (2B8) PE Cy5 (ebioscience) and 1 μl/ml Fixable viability dye eFluor 450 (ebioscience) and incubated for 40 min at 4°C in the dark. OneComp eBeads (ebioscience) were used for single stained controls. Cells were washed in FACs buffer, resuspended in DMEM with 25 mM HEPES at a concentration of 1 x 10^7^ cells/ml and filtered through a 50‐μm filter (BD Biosciences). The cells were sorted into immature B cells (B220 ^+^ IgM ^+^ IgD^‐^), mature B cells (B220 ^+^ IgM ^+^ IgD^+^), pro B cells (B220 ^+^ IgM^‐^IgD^‐^C‐kit^+^) and pre B cells (B220 ^+^ IgM^‐^IgD^‐^C‐kit^‐^) using a FACS Aria (BD Bioscience) and collected into a 5 ml polypropylene tube containing DMEM with 10% FBS.

### 4.15 *Hoxc13* and *Spi1* RT‐PCR

RNA was prepared from mouse E16.5 fetal liver, adult bone marrow, E16.5 hind‐limb, and adult spleen tissue samples, and FACS isolated B‐cell fractions, with TRI‐Reagent (Sigma), treated with DNase, and reverse‐transcribed with BioScript RT (Bioline). PCR primers are listed in Supporting Information Table 2. Cycling conditions were: 95°C 4 min, and then followed by 95°C 45 s, 58°C (*Hoxc13*) or 60°C (*Spi1*) 45 s and 72°C 1 min for 35 cycles, and 72°C 10 min.

### 4.16 *Zfp521*, *Spi1*, and *Hoxc13* quantitative RT‐PCR

BCL1 or BA/F3 cells were transfected with pFlagCMV‐HOXC13 and pCMVSport6‐Spi1 (Amaxa nucleofector), or empty expression vector control, and incubated for 2 days prior to RNA harvesting. Foetal livers were dissected from Tg(Hoxc13)^61B1Awg^ E16.5 transgenic mice or nontransgenic littermate controls. All animal work at the Medical University of South Carolina was done in compliance with MUSC institutional animal care and used committee‐approved procedures. Total RNA was extracted with TRI reagent (Sigma), treated with DNaseI, reverse transcribed, and subjected to real‐time quantitative PCR for *Zfp521* and *Gapdh* (Eurogentec qPCR Core kit for SYBR Green). *Zfp521* primers were previously reported (Hentges et al., 2005). *Hoxc13* primers sequences were the same as those used for RT‐PCR. *Gapdh* primer sequences listed in Supporting Information Table 2. Reaction conditions were 95°C for 10 minutes, followed by 95°C 15 seconds, 60°C 1 minute for 40 cycles. Expression levels were normalized to *Gapdh*.

## Supporting information

Supporting Information Figure 1

Supporting Information Table 1

Supporting Information Table 2

## References

[dvg22963-bib-0001] Addison, W. N. , Fu, M. M. , Yang, H. X. , Lin, Z. , Nagano, K. , Gori, F. , & Baron, R. (2014). Direct transcriptional repression of Zfp423 by Zfp521 mediates a bone morphogenic protein‐dependent osteoblast versus adipocyte lineage commitment switch. Mol Cell Biol, 34, 3076–3085. 24891617 10.1128/MCB.00185-14 PMC4135594

[dvg22963-bib-0002] Al Dallal, S. , Wolton, K. , & Hentges, K. E. (2016). Zfp521 promotes B‐cell viability and cyclin D1 gene expression in a B cell culture system. Leuk Res, 46, 10–17. 27107743 10.1016/j.leukres.2016.03.013 PMC4910839

[dvg22963-bib-0003] Aoki, Y. , Watanabe, T. , Saito, Y. , Kuroki, Y. , Hijikata, A. , Takagi, M. , … Ishikawa, F. (2015). Identification of CD34+ and CD34‐ leukemia‐initiating cells in MLL‐rearranged human acute lymphoblastic leukemia. Blood, 125, 967–980. 25538041 10.1182/blood-2014-03-563304 PMC4319237

[dvg22963-bib-0004] Argiropoulos, B. , & Humphries, R. K. (2007). Hox genes in hematopoiesis and leukemogenesis. Oncogene, 26, 6766–6776. 17934484 10.1038/sj.onc.1210760

[dvg22963-bib-0005] Bijl, J. , Sauvageau, M. , Thompson, A. , & Sauvageau, G. (2005). High incidence of proviral integrations in the Hoxa locus in a new model of E2a‐PBX1‐induced B‐cell leukemia. Genes Dev, 19, 224–233. 15655112 10.1101/gad.1268505 PMC545883

[dvg22963-bib-0006] Busslinger, M. (2004). Transcriptional control of early B cell development. Annu Rev Immunol, 22, 55–79. 15032574 10.1146/annurev.immunol.22.012703.104807

[dvg22963-bib-0007] Chen, F. , Li, Y. , Wang, L. , & Hu, L. (2011). Knockdown of BMI‐1 causes cell‐cycle arrest and derepresses p16INK4a, HOXA9 and HOXC13 mRNA expression in HeLa cells. Med Oncol, 28, 1201–1209. 20661663 10.1007/s12032-010-9634-9

[dvg22963-bib-0008] Cheng, L. E. , & Reed, R. R. (2007). Zfp423/OAZ participates in a developmental switch during olfactory neurogenesis. Neuron, 54, 547–557. 17521568 10.1016/j.neuron.2007.04.029 PMC2866517

[dvg22963-bib-0009] Correa, D. , Hesse, E. , Seriwatanachai, D. , Kiviranta, R. , Saito, H. , Yamana, K. , … Baron, R. (2010). Zfp521 is a target gene and key effector of parathyroid hormone‐related peptide signaling in growth plate chondrocytes. Dev Cell, 19, 533–546. 20951345 10.1016/j.devcel.2010.09.008 PMC2958174

[dvg22963-bib-0010] DeKoter, R. P. , Lee, H. J. , & Singh, H. (2002). PU.1 regulates expression of the interleukin‐7 receptor in lymphoid progenitors. Immunity, 16, 297–309. 11869689 10.1016/s1074-7613(02)00269-8

[dvg22963-bib-0011] Dias, S. , Xu, W. , McGregor, S. , & Kee, B. (2008). Transcriptional regulation of lymphocyte development. Curr Opin Genet Dev, 18, 441–448. 18775782 10.1016/j.gde.2008.07.015 PMC2743889

[dvg22963-bib-0012] Edgar, R. C. (2004). MUSCLE: multiple sequence alignment with high accuracy and high throughput. Nucleic Acids Res, 32, 1792–1797. 15034147 10.1093/nar/gkh340 PMC390337

[dvg22963-bib-0013] Gupta, R. K. , Arany, Z. , Seale, P. , Mepani, R. J. , Ye, L. , Conroe, H. M. , … Spiegelman, B. M. (2010). Transcriptional control of preadipocyte determination by Zfp423. Nature, 464, 619–623. 20200519 10.1038/nature08816 PMC2845731

[dvg22963-bib-0014] Gupta, R. K. , Mepani, R. J. , Kleiner, S. , Lo, J. C. , Khandekar, M. J. , Cohen, P. , … Spiegelman, B. M. (2012). Zfp423 expression identifies committed preadipocytes and localizes to adipose endothelial and perivascular cells. Cell Metab, 15, 230–239. 22326224 10.1016/j.cmet.2012.01.010 PMC3366493

[dvg22963-bib-0015] Han, R. , Kan, Q. , Sun, Y. , Wang, S. , Zhang, G. , Peng, T. , & Jia, Y. (2012). MiR‐9 promotes the neural differentiation of mouse bone marrow mesenchymal stem cells via targeting zinc finger protein 521. Neurosci Lett, 515, 147–152. 22465325 10.1016/j.neulet.2012.03.032

[dvg22963-bib-0016] Hashemi, M. S. , Esfahani, A. K. , Peymani, M. , Nejati, A. S. , Ghaedi, K. , Nasr‐Esfahani, M. H. , & Baharvand, H. (2016). Zinc finger protein 521 overexpression increased transcript levels of Fndc5 in mouse embryonic stem cells. J Biosci, 41, 69–76. 26949089 10.1007/s12038-015-9578-5

[dvg22963-bib-0017] Hegde, S. , Ni, S. , He, S. , Yoon, D. , Feng, G. S. , Watowich, S. S. , … Hankey, P. A. (2009). Stat3 promotes the development of erythroleukemia by inducing Pu.1 expression and inhibiting erythroid differentiation. Oncogene, 28, 3349–3359. 19581930 10.1038/onc.2009.202 PMC3086737

[dvg22963-bib-0018] Hentges, K. E. , Weiser, K. C. , Schountz, T. , Woodward, L. S. , Morse, H. C. , & Justice, M. J. (2005). Evi3, a zinc‐finger protein related to EBFAZ, regulates EBF activity in B‐cell leukemia. Oncogene, 24, 1220–1230. 15580294 10.1038/sj.onc.1208243

[dvg22963-bib-0019] Hesse, E. , Kiviranta, R. , Wu, M. , Saito, H. , Yamana, K. , Correa, D. , … Baron, R. (2010). Zinc finger protein 521, a new player in bone formation. Ann N Y Acad Sci, 1192, 32–37. 20392215 10.1111/j.1749-6632.2009.05347.x

[dvg22963-bib-0020] Hiratsuka, T. , Takei, Y. , Ohmori, R. , Imai, Y. , Ozeki, M. , Tamaki, K. , … Tsuruyama, T. (2015). ZFP521 contributes to pre‐B‐cell lymphomagenesis through modulation of the pre‐B‐cell receptor signaling pathway. Oncogene. 35, 3227–3238. 26522721 10.1038/onc.2015.385

[dvg22963-bib-0021] Ho, L. , & Crabtree, G. R. (2010). Chromatin remodelling during development. Nature, 463, 474–484. 20110991 10.1038/nature08911 PMC3060774

[dvg22963-bib-0022] Jacobs, J. J. , Scheijen, B. , Voncken, J. W. , Kieboom, K. , Berns, A. , & van Lohuizen, M. (1999). Bmi‐1 collaborates with c‐Myc in tumorigenesis by inhibiting c‐Myc‐induced apoptosis via INK4a/ARF. Genes Dev, 13, 2678–2690. 10541554 10.1101/gad.13.20.2678 PMC317101

[dvg22963-bib-0023] Kamiya, D. , Banno, S. , Sasai, N. , Ohgushi, M. , Inomata, H. , Watanabe, K. , … Sasai, Y. (2011). Intrinsic transition of embryonic stem‐cell differentiation into neural progenitors. Nature, 470, 503–509. 21326203 10.1038/nature09726

[dvg22963-bib-0024] Kang, S. , Akerblad, P. , Kiviranta, R. , Gupta, R. K. , Kajimura, S. , Griffin, M. J. , … Rosen, E. D. (2012). Regulation of early adipose commitment by Zfp521. PLoS Biol, 10, e1001433. 23209378 10.1371/journal.pbio.1001433 PMC3507953

[dvg22963-bib-0025] Kiviranta, R. , Yamana, K. , Saito, H. , Ho, D. K. , Laine, J. , Tarkkonen, K. , … Baron, R. (2013). Coordinated transcriptional regulation of bone homeostasis by Ebf1 and Zfp521 in both mesenchymal and hematopoietic lineages. J Exp Med, 210, 969–985. 23569325 10.1084/jem.20121187 PMC3646489

[dvg22963-bib-0026] Ko, K. H. , Lam, Q. L. , Zhang, M. , Wong, C. K. , Lo, C. K. , Kahmeyer‐Gabbe, M. , … Lu, L. (2007). Hoxb3 deficiency impairs B lymphopoiesis in mouse bone marrow. Exp Hematol, 35, 465–475. 17309827 10.1016/j.exphem.2006.10.014

[dvg22963-bib-0027] Kodandapani, R. , Pio, F. , Ni, C. Z. , Piccialli, G. , Klemsz, M. , McKercher, S. , … Ely, K. R. (1996). A new pattern for helix‐turn‐helix recognition revealed by the PU.1 ETS‐domain‐DNA complex. Nature, 380, 456–460. 8602247 10.1038/380456a0

[dvg22963-bib-0028] Lawrence, H. J. , Helgason, C. D. , Sauvageau, G. , Fong, S. , Izon, D. J. , Humphries, R. K. , & Largman, C. (1997). Mice bearing a targeted interruption of the homeobox gene HOXA9 have defects in myeloid, erythroid, and lymphoid hematopoiesis. Blood, 89, 1922–1930. 9058712

[dvg22963-bib-0029] Li, R. , Pei, H. , & Watson, D. K. (2000). Regulation of Ets function by protein–protein interactions. Oncogene, 19, 6514–6523. 11175367 10.1038/sj.onc.1204035

[dvg22963-bib-0030] Lobo, M. K. , Karsten, S. L. , Gray, M. , Geschwind, D. H. , & Yang, X. W. (2006). FACS‐array profiling of striatal projection neuron subtypes in juvenile and adult mouse brains. Nat Neurosci, 9, 443–452. 16491081 10.1038/nn1654

[dvg22963-bib-0031] Matsubara, E. , Sakai, I. , Yamanouchi, J. , Fujiwara, H. , Yakushijin, Y. , Hato, T. , … Yasukawa, M. (2009). The role of zinc finger protein 521/early hematopoietic zinc finger protein in erythroid cell differentiation. J Biol Chem, 284, 3480–3487. 19049973 10.1074/jbc.M805874200

[dvg22963-bib-0032] Medina, K. L. , & Singh, H. (2005). Genetic networks that regulate B lymphopoiesis. Curr Opin Hematol, 12, 203–209. 15867576 10.1097/01.moh.0000160735.67596.a0

[dvg22963-bib-0033] Mega, T. , Lupia, M. , Amodio, N. , Horton, S. J. , Mesuraca, M. , Pelaggi, D. , … Morrone, G. (2011). Zinc finger protein 521 antagonizes early B‐cell factor 1 and modulates the B‐lymphoid differentiation of primary hematopoietic progenitors. Cell Cycle, 10, 2129–2139. 21593590 10.4161/cc.10.13.16045

[dvg22963-bib-0034] Mesuraca, M. , Chiarella, E. , Scicchitano, S. , Codispoti, B. , Giordano, M. , Nappo, G. , … Morrone, G. (2015). ZNF423 and ZNF521: EBF1 Antagonists of Potential Relevance in B‐Lymphoid Malignancies. Biomed Res Int, 2015, 165238. 26788497 10.1155/2015/165238 PMC4695665

[dvg22963-bib-0035] Mullighan, C. G. , Goorha, S. , Radtke, I. , Miller, C. B. , Coustan‐Smith, E. , Dalton, J. D. , … Downing, J. R. (2007). Genome‐wide analysis of genetic alterations in acute lymphoblastic leukaemia. Nature, 446, 758–764. 17344859 10.1038/nature05690

[dvg22963-bib-0036] Northrup, D. L. , & Allman, D. (2008). Transcriptional regulation of early B cell development. Immunol Res, 42, 106–117. 18818886 10.1007/s12026-008-8043-z

[dvg22963-bib-0037] Nutt, S. L. , & Kee, B. L. (2007). The transcriptional regulation of B cell lineage commitment. Immunity, 26, 715–725. 17582344 10.1016/j.immuni.2007.05.010

[dvg22963-bib-0038] Ohkubo, N. , Matsubara, E. , Yamanouchi, J. , Akazawa, R. , Aoto, M. , Suzuki, Y. , … Mitsuda, N. (2014). Abnormal behaviors and developmental disorder of hippocampus in zinc finger protein 521 (ZFP521) mutant mice. PLoS One, 9, e92848. 24676388 10.1371/journal.pone.0092848 PMC3968043

[dvg22963-bib-0039] Oikawa, T. , Yamada, T. , Kihara‐Negishi, F. , Yamamoto, H. , Kondoh, N. , Hitomi, Y. , & Hashimoto, Y. (1999). The role of Ets family transcription factor PU.1 in hematopoietic cell differentiation, proliferation and apoptosis. Cell Death Differ, 6, 599–608. 10453070 10.1038/sj.cdd.4400534

[dvg22963-bib-0040] Pang, S. H. , Minnich, M. , Gangatirkar, P. , Zheng, Z. , Ebert, A. , Song, G. , … Carotta, S. (2016). PU.1 cooperates with IRF4 and IRF8 to suppress pre‐B‐cell leukemia. Leukemia 30, 1375–1387. 26932576 10.1038/leu.2016.27 PMC5179358

[dvg22963-bib-0041] Park, S. Y. , & Kim, J. E. (2013). Differential gene expression by Osterix knockdown in mouse chondrogenic ATDC5 cells. Gene, 518, 368–375. 23337593 10.1016/j.gene.2012.12.102

[dvg22963-bib-0042] Potter, C. S. , Peterson, R. L. , Barth, J. L. , Pruett, N. D. , Jacobs, D. F. , Kern, M. J. , … Awgulewitsch, A. (2006). Evidence that the satin hair mutant gene Foxq1 is among multiple and functionally diverse regulatory targets for Hoxc13 during hair follicle differentiation. J Biol Chem, 281, 29245–29255. 16835220 10.1074/jbc.M603646200

[dvg22963-bib-0043] Sabo, P. J. , Kuehn, M. S. , Thurman, R. , Johnson, B. E. , Johnson, E. M. , Cao, H. , … Stamatoyannopoulos, J. A. (2006). Genome‐scale mapping of DNase I sensitivity in vivo using tiling DNA microarrays. Nat Methods, 3, 511–518. 16791208 10.1038/nmeth890

[dvg22963-bib-0044] Sauvageau, G. , Thorsteinsdottir, U. , Hough, M. R. , Hugo, P. , Lawrence, H. J. , Largman, C. , & Humphries, R. K. (1997). Overexpression of HOXB3 in hematopoietic cells causes defective lymphoid development and progressive myeloproliferation. Immunity, 6, 13–22. 9052833 10.1016/s1074-7613(00)80238-1

[dvg22963-bib-0045] Schug, J. 2008 Using TESS to Predict Transcription Factor Binding Sites in DNA Sequence. Wiley, *Curr Protoc Bioinformatics*, doi:10.1002/0471250953.bi0206s21. 10.1002/0471250953.bi0206s21 18428685

[dvg22963-bib-0046] Scott, E. W. , Simon, M. C. , Anastasi, J. , & Singh, H. (1994). Requirement of transcription factor PU.1 in the development of multiple hematopoietic lineages. Science, 265, 1573–1577. 8079170 10.1126/science.8079170

[dvg22963-bib-0047] Singh, H. , Medina, K. L. , & Pongubala, J. M. (2005). Contingent gene regulatory networks and B cell fate specification. Proc Natl Acad Sci USA, 102, 4949–4953. 15788530 10.1073/pnas.0500480102 PMC555998

[dvg22963-bib-0048] Sive, J. I. , Basilico, S. , Hannah, R. , Kinston, S. J. , Calero‐Nieto, F. J. , & Gottgens, B. (2016). Genome‐scale definition of the transcriptional programme associated with compromised PU.1 activity in acute myeloid leukaemia. Leukemia, 30, 14–23. 26126967 10.1038/leu.2015.172 PMC4705427

[dvg22963-bib-0049] Sokalski, K. M. , Li, S. K. , Welch, I. , Cadieux‐Pitre, H. A. , Gruca, M. R. , & DeKoter, R. P. (2011). Deletion of genes encoding PU.1 and Spi‐B in B cells impairs differentiation and induces pre‐B cell acute lymphoblastic leukemia. Blood, 118, 2801–2808. 21768304 10.1182/blood-2011-02-335539

[dvg22963-bib-0050] Spina, R. , Filocamo, G. , Iaccino, E. , Scicchitano, S. , Lupia, M. , Chiarella, E. , … Morrone, G. (2013). Critical role of zinc finger protein 521 in the control of growth, clonogenicity and tumorigenic potential of medulloblastoma cells. Oncotarget, 4, 1280–1292. 23907569 10.18632/oncotarget.1176 PMC3787157

[dvg22963-bib-0051] Tang, K. , Peng, G. , Qiao, Y. , Song, L. , & Jing, N. (2015). Intrinsic regulations in neural fate commitment. Dev Growth Differ, 57, 109–120. 25708399 10.1111/dgd.12204

[dvg22963-bib-0052] Tkatchenko, A. V. , Visconti, R. P. , Shang, L. , Papenbrock, T. , Pruett, N. D. , Ito, T. , … Awgulewitsch, A. (2001). Overexpression of Hoxc13 in differentiating keratinocytes results in downregulation of a novel hair keratin gene cluster and alopecia. Development, 128, 1547–1558. 11290294 10.1242/dev.128.9.1547

[dvg22963-bib-0053] Vonlanthen, S. , Heighway, J. , Altermatt, H. J. , Gugger, M. , Kappeler, A. , Borner, M. M. , … Betticher, D. C. (2001). The bmi‐1 oncoprotein is differentially expressed in nonsmall cell lung cancer and correlates with INK4A‐ARF locus expression. Br J Cancer, 84, 1372–1376. 11355949 10.1054/bjoc.2001.1791 PMC2363629

[dvg22963-bib-0054] Warming, S. , Liu, P. , Suzuki, T. , Akagi, K. , Lindtner, S. , Pavlakis, G. N. , … Copeland, N. G. (2003). Evi3, a common retroviral integration site in murine B‐cell lymphoma, encodes an EBFAZ‐related Kruppel‐like zinc finger protein. Blood, 101, 1934–1940. 12393497 10.1182/blood-2002-08-2652

[dvg22963-bib-0055] Warming, S. , Rachel, R. A. , Jenkins, N. A. , & Copeland, N. G. (2006). Zfp423 is required for normal cerebellar development. Mol Cell Biol, 26, 6913–6922. 16943432 10.1128/MCB.02255-05 PMC1592861

[dvg22963-bib-0056] Warming, S. , Suzuki, T. , Yamaguchi, T. P. , Jenkins, N. A. , & Copeland, N. G. (2004). Early B‐cell factor‐associated zinc‐finger gene is a frequent target of retroviral integration in murine B‐cell lymphomas. Oncogene, 23, 2727–2731. 15048087 10.1038/sj.onc.1207452

[dvg22963-bib-0057] Will, B. , Vogler, T. O. , Narayanagari, S. , Bartholdy, B. , Todorova, T. I. , da Silva Ferreira, M. , … Steidl, U. (2015). Minimal PU.1 reduction induces a preleukemic state and promotes development of acute myeloid leukemia. Nat Med, 21, 1172–1181. 26343801 10.1038/nm.3936 PMC5144917

[dvg22963-bib-0058] Yamada, T. , Shimizu, T. , Sakurai, T. , Nanashima, N. , Kihara‐Negishi, F. , Suzuki, M. , … Tsuchida, S. (2009). Physical and functional interactions between hematopoietic cell‐specific ETS transcription factors and homeodomain proteins. Leuk Res, 33, 483–489. 18692240 10.1016/j.leukres.2008.07.002

[dvg22963-bib-0059] Yamada, T. , Shimizu, T. , Suzuki, M. , Kihara‐Negishi, F. , Nanashima, N. , Sakurai, T. , … Tsuchida, S. (2008). Interaction between the homeodomain protein HOXC13 and ETS family transcription factor PU.1 and its implication in the differentiation of murine erythroleukemia cells. Exp Cell Res, 314, 847–858. 18076876 10.1016/j.yexcr.2007.11.005

[dvg22963-bib-0060] Yamasaki, N. , Miyazaki, K. , Nagamachi, A. , Koller, R. , Oda, H. , Miyazaki, M. , … Honda, H. (2010). Identification of Zfp521/ZNF521 as a cooperative gene for E2A‐HLF to develop acute B‐lineage leukemia. Oncogene, 29, 1963–1975. 20062079 10.1038/onc.2009.475

[dvg22963-bib-0061] Yang, H. , Liang, H. , Yan, J. S. , Tao, R. , Hao, S. G. , & Ma, L. Y. (2012). Down‐regulation of hematopoiesis master regulator PU.1 via aberrant methylation in chronic myeloid leukemia. Int J Hematol, 96, 65–73. 22674382 10.1007/s12185-012-1106-x

[dvg22963-bib-0062] Yokota, T. , Huang, J. , Tavian, M. , Nagai, Y. , Hirose, J. , Zuniga‐Pflucker, J. C. , … Kincade, P. W. (2006). Tracing the first waves of lymphopoiesis in mice. Development, 133, 2041–2051. 16611687 10.1242/dev.02349

[dvg22963-bib-0063] Zhou, J. , Wu, J. , Li, B. , Liu, D. , Yu, J. , Yan, X. , … Wang, Q. F. (2014). PU.1 is essential for MLL leukemia partially via crosstalk with the MEIS/HOX pathway. Leukemia, 28, 1436–1448. 24445817 10.1038/leu.2013.384 PMC4410691

