# Supplementary figures and images for: Transcriptional regulation of the proto‐oncogene Zfp521 by SPI1 (PU.1) and HOXC13
Source: Genesis. 2016 Aug 29;54(10):519–33. doi: 10.1002/dvg.22963 (PMC5073027; doi:10.1002/dvg.22963)

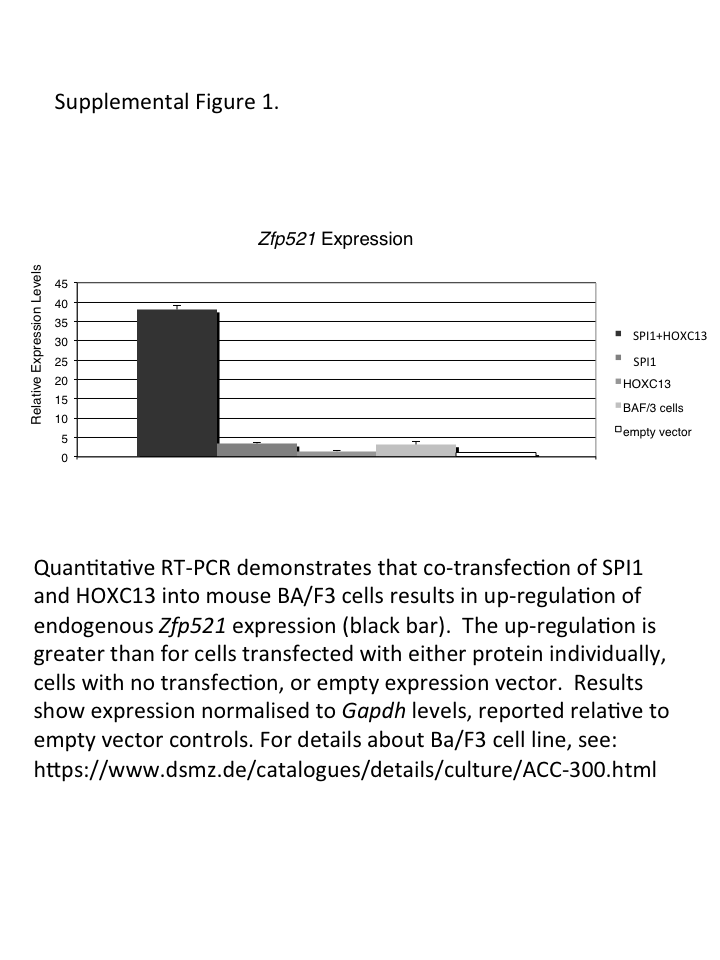

Supplement: Supplementary file 1 — Supporting Information Figure 1 [file DVG-54-519-s001.tif]
