# Supplementary material for: Transcriptional regulation of the proto‐oncogene Zfp521 by SPI1 (PU.1) and HOXC13
Source: Genesis. 2016 Aug 29;54(10):519–33. doi: 10.1002/dvg.22963 (PMC5073027; doi:10.1002/dvg.22963)

Supplemental Table 1. ZNF521 and ZNF423 protein sequences used in phylogeny analysis, and schematic of phylogenetic analysis protocol.

| **Species** | **Zfp521** | **Zfp423** | **B-cells present?** |
| --- | --- | --- | --- |
| Human (Homo sapiens) | [NP_056276](http://www.ncbi.nlm.nih.gov/entrez/viewer.fcgi?db=protein&val=24308069) (Hu521 6276)  [AAI13623](http://www.ncbi.nlm.nih.gov/entrez/viewer.fcgi?db=protein&id=109731099)  [CAD57322](http://www.ncbi.nlm.nih.gov/entrez/viewer.fcgi?db=protein&val=25264930)  [BAB55056](http://www.ncbi.nlm.nih.gov/entrez/viewer.fcgi?db=protein&val=14041974)  [AAI13649](http://www.ncbi.nlm.nih.gov/entrez/viewer.fcgi?db=protein&val=109731353)  [EAX01201](http://www.ncbi.nlm.nih.gov/entrez/viewer.fcgi?db=protein&val=119621606)  [BAB84872](http://www.ncbi.nlm.nih.gov/entrez/viewer.fcgi?db=protein&val=18676440)  [AAH32869](http://www.ncbi.nlm.nih.gov/entrez/viewer.fcgi?db=protein&val=34192845) | [NP_055884](http://www.ncbi.nlm.nih.gov/entrez/viewer.fcgi?db=protein&id=46359075) (Human423 E)  [AAF28354](http://www.ncbi.nlm.nih.gov/entrez/viewer.fcgi?db=protein&id=6760445)  [AAI12318](http://www.ncbi.nlm.nih.gov/entrez/viewer.fcgi?db=protein&val=85567335)  [AAI12316](http://www.ncbi.nlm.nih.gov/entrez/viewer.fcgi?db=protein&val=85567729)  [BAA34480](http://www.ncbi.nlm.nih.gov/entrez/viewer.fcgi?db=protein&val=20521644) | Y |
| Chimp (Pan troglodytes) | [XP_512068](http://www.ncbi.nlm.nih.gov/entrez/viewer.fcgi?val=114672766) (Pan521 068) | [XP_001163978](http://www.ncbi.nlm.nih.gov/entrez/viewer.fcgi?val=114662385) (Pan423 978)  [XP_520629](http://www.ncbi.nlm.nih.gov/entrez/viewer.fcgi?db=protein&val=114662387) | Y |
| Rhesus (Macaca mulatta) | [XP_001097677](http://www.ncbi.nlm.nih.gov/entrez/viewer.fcgi?db=protein&id=109121833) (Mac521 677) | [XP_001082507](http://www.ncbi.nlm.nih.gov/entrez/viewer.fcgi?db=protein&id=109128453) (Mac423 507)  [XP_001082640](http://www.ncbi.nlm.nih.gov/entrez/viewer.fcgi?db=protein&val=109128455) | Y |
| Mouse (Mus musculus) | [NP_663467](http://www.ncbi.nlm.nih.gov/entrez/viewer.fcgi?db=protein&id=21703968) (Mu521 3467)  [AAN39839](http://www.ncbi.nlm.nih.gov/entrez/viewer.fcgi?val=24061769)  [BAD21380](http://www.ncbi.nlm.nih.gov/entrez/viewer.fcgi?db=protein&val=47847416)  [AAH21376](http://www.ncbi.nlm.nih.gov/entrez/viewer.fcgi?db=protein&val=18204060)  [EDL01581](http://www.ncbi.nlm.nih.gov/entrez/viewer.fcgi?db=protein&val=148669634)  [EDL01582](http://www.ncbi.nlm.nih.gov/entrez/viewer.fcgi?db=protein&val=148669635) | [NP_201584](http://www.ncbi.nlm.nih.gov/entrez/viewer.fcgi?val=46359077) (Mu423 1584)  [AAP33073](http://www.ncbi.nlm.nih.gov/entrez/viewer.fcgi?val=37955660)  [AAN39840](http://www.ncbi.nlm.nih.gov/entrez/viewer.fcgi?val=24061772)  [AAG17053](http://www.ncbi.nlm.nih.gov/entrez/viewer.fcgi?val=10441461)  [BAC65647](http://www.ncbi.nlm.nih.gov/entrez/viewer.fcgi?db=protein&val=28972387)  [XP_001000788](http://www.ncbi.nlm.nih.gov/entrez/viewer.fcgi?db=protein&val=94384211)  [AAH59234](http://www.ncbi.nlm.nih.gov/entrez/viewer.fcgi?db=protein&val=37589230)  [EDL11043](http://www.ncbi.nlm.nih.gov/entrez/viewer.fcgi?db=protein&val=148679096)  [XP_001000781](http://www.ncbi.nlm.nih.gov/entrez/viewer.fcgi?db=protein&val=94384213)  [XP_001000774](http://www.ncbi.nlm.nih.gov/entrez/viewer.fcgi?db=protein&val=94384215)  [EDL11044](http://www.ncbi.nlm.nih.gov/entrez/viewer.fcgi?db=protein&val=148679097)  [BAE21688](http://www.ncbi.nlm.nih.gov/entrez/viewer.fcgi?db=protein&val=74225718) | Y |
| Rat (Rattus norvegicus) | [EDL75029](http://www.ncbi.nlm.nih.gov/entrez/viewer.fcgi?val=149015670)  [XP_001073019](http://www.ncbi.nlm.nih.gov/entrez/viewer.fcgi?db=protein&val=109506968) (Rat521 019)  [EDL75030](http://www.ncbi.nlm.nih.gov/entrez/viewer.fcgi?db=protein&val=149015671)  [XP_226153](http://www.ncbi.nlm.nih.gov/entrez/viewer.fcgi?val=XM_226153.4) | [EDL87518](http://www.ncbi.nlm.nih.gov/entrez/viewer.fcgi?val=149032648)  [NP_446035](http://www.ncbi.nlm.nih.gov/entrez/viewer.fcgi?db=protein&val=16758356) (Rat423 035)  [AAB58646](http://www.ncbi.nlm.nih.gov/entrez/viewer.fcgi?db=protein&val=2149792) | Y |
| Cat (Felis catus) |  |  | Y |
| Dog (Canis familiaris) | [XP_547633](http://www.ncbi.nlm.nih.gov/entrez/viewer.fcgi?val=73961326) (Dog521 633)  [XP_866970](http://www.ncbi.nlm.nih.gov/entrez/viewer.fcgi?db=protein&val=73961328) | [XP_544417](http://www.ncbi.nlm.nih.gov/entrez/viewer.fcgi?val=73950414) (Dog423 417) | Y |
| Cow (Bos taurus) | [XP_582772](http://www.ncbi.nlm.nih.gov/entrez/viewer.fcgi?val=119916162) (Cow521 772) | [XP_876109](http://www.ncbi.nlm.nih.gov/entrez/viewer.fcgi?val=119910017) (Cow423 109) | Y |
| Opossum (Monodelphis domestica) | [XP_001364817](http://www.ncbi.nlm.nih.gov/entrez/viewer.fcgi?val=126321771) (Op521 4817) | [XP_001371030](http://www.ncbi.nlm.nih.gov/entrez/viewer.fcgi?val=126296289) (Op423 1030) | Y |
| Chicken (Gallus gallus) | [XP_419167](http://www.ncbi.nlm.nih.gov/entrez/viewer.fcgi?val=118086914) (Gal521 167) | [XP_414103](http://www.ncbi.nlm.nih.gov/entrez/viewer.fcgi?val=118096326) (Gal423103) | Y |
| Frog (Xenopus laevis) | [NP_001084597](http://www.ncbi.nlm.nih.gov/entrez/viewer.fcgi?val=147905462)  [AAH68658](http://www.ncbi.nlm.nih.gov/entrez/viewer.fcgi?db=protein&val=46250062) | NP_001159911 | Y |
| Zebrafish (Danio rerio) | [XP_700319](http://www.ncbi.nlm.nih.gov/entrez/viewer.fcgi?val=125843593) (Dan521 319) | [NP_001073499](http://www.ncbi.nlm.nih.gov/entrez/viewer.fcgi?val=121583818) (Dan423 499)  [AAI29185](http://www.ncbi.nlm.nih.gov/entrez/viewer.fcgi?db=protein&val=120537605)  [CAK04518](http://www.ncbi.nlm.nih.gov/entrez/viewer.fcgi?db=protein&val=94732648) | Y |
| Fugu (Takifugu rubripes) | Potential ZNF521 | [CAAB01003300](http://www.ncbi.nlm.nih.gov/entrez/viewer.fcgi?db=nucleotide&val=22421363)  Gap in sequence | Y |
| Stickleback (Gasterosteus aculeatus) | Potential ZNF521 | [AANH01008668](http://www.ncbi.nlm.nih.gov/entrez/viewer.fcgi?db=nucleotide&val=86294020)  Gap in sequence | Y |
| SeaUrchin (Strongylocentrotus purpuratis) | strPur1_dna range=Scaffold109473:17367-22190 | | N |
| Drosophila melanogaster | [NP_610967](http://www.ncbi.nlm.nih.gov/entrez/viewer.fcgi?db=protein&val=20129991) (Drosophila) | | N |
| HoneyBee (Apis mellifera) | [XP_396910](http://www.ncbi.nlm.nih.gov/entrez/viewer.fcgi?db=protein&val=110762676) (Honeybee g) | | N |
| Anopheles gambiea | [EAA05280](http://www.ncbi.nlm.nih.gov/entrez/viewer.fcgi?db=protein&val=116131769) (Anopheles) | | N |
| Beetle (Tribolium castaneum) | [XP_966615](http://www.ncbi.nlm.nih.gov/entrez/viewer.fcgi?db=protein&val=91075920) (Beetle gi) | | N |

>Gal521|167gi|118086914|ref|XP_419167.2| PREDICTED: similar to ZNF521 protein [Gallus gallus]

MTNSCSTETQPGNKGIPALRPRPVRPRAPDLALCPVREAAPCTSWQHWGISSRPQLAISSCLELGEPSTT

LEPAAPEPKPWLCHSCPFDSRQADNDFLSDYVQSESLRVRGPLWSHPDGFGGLASIFHYYVDYRSDSAVQ

DLADPNCKLEDKTEDGEVLDCKKRPDEGEELEEEAVHSCDSCLQVFESLSDITEHKINQCQLTDGVDVED

DPTCSWPASSPSSKDQTSPSHGEGCDFGEEEGGPGLPYQCQFCDKSFSRLSYLKHHEQSHSDKLPFKCTY

CSRLFKHKRSRDRHIKLHTGDKKYHCSECDAAFSRSDHLKIHLKTHTSNKPYKCAICRRGFLSSSSLHGH

MQVHERNKDGSQSASRMEDWKMKDTQKCSQCEEGFDFPEDLQKHIAECHPECSPNEDRSALQCVYCHELF

VEETSLVNHMEQAHNGEKKNSCSICSENFHTVEELYSHMDSHQQPESCNHSNSPSLVTVGYTSVSSTTPD

SNLSVDSSTMVETAPPIPKGRGRKRAAQQVPDITGPSSKQAKVTYSCIYCNKQLFSSLAVLQIHLKTMHL

DKPEQAHICQYCLEVLPSLYNLNEHLKQVHEAPDPGLIVSTMPAMVYQCNFCSEVFNDLNTLQEHIRCSH

GFANPAAKDSNAFFCPHCYMGFLTDSSLEEHIRQVHCDLSSSRFGSPVLGTPKDPVVEVYSCSYCTNSPI

FNSVLKLNKHIKENHKNIPLALNYIHNGKKSRAMSPLSPVTIEQTSLKMMQAVGGAPPRPAGEYICNQCG

AKYTSLDGFQTHLKTHLDTVLPKLTCPQCNKEFPNQESLLKHVTIHFMITSTYYICESCDKQFTSVDDLQ

KHLLDMHTFVFFRCTLCQEVFDSKVSIQLHLAVKHSNEKKVYRCTSCNWDFRNETDLQLHVKHNHLENQG

KVHKCIFCGESFGTEVELQCHITTHSKKYNCKFCSKAFHAIILLEKHLREKHCVFETKTPNCGTNGASEQ

VQKEEVELQTLLTNSQESHNSHDGSEEDVDTSEPMYGCDICGAAYTMETLLQNHQLRDHNIRPGESAIVK

KKAELIKGNYKCNVCSRTFFSENGLREHMQTHLGPVKHYMCPICGERFPSLLTLTEHKVTHSKSLDTGNC

RICKMPLQSEEEFLEHCQMHPDLRNSLTGFRCVVCMQTVTSTLELKIHGTFHMQKTGNGSAVQSTGRAQH

LQKLYKCASCLKEFRSKQDLVKLDINGLPYGLCASCVNLSKSGSPSVNIPSSSNRQGMGQNENLSSIENK

SKAGGLKTRCSSCNVKFESESELQNHIQSIHRELVPDSNSTQLKTPQVSPMPRISPSQTEEKKTYQCIKC

QMVFYNEWDIQVHVANHMIDEGLNHECKLCNQTFDSPAKLQCHLIEHSFEGMGGTFKCPVCFTVFVQANK

LQQHIFSAHGQEDKIYDCTQCPQKFFFQTELQNHTMTQHSS

>Pan521|068gi|114672766|ref|XP_512068.2| PREDICTED: hypothetical protein [Pan troglodytes]

MSRRKQAKPRSLKDPNCKLEDKTEDGEALDCKKRPEDGEELEDEAVHSCDSCLQVFESLSDITEHKINQC

QLTDGVDVEDDPTCSWPASSPSSKDQTSPSHGEGCDFGEEEGGPGLPYPCQFCDKSFSRLSYLKHHEQSH

SDKLPFKCTYCSRLFKHKRSRDRHIKLHTGDKKYHCSECDAAFSRSDHLKIHLKTHTSNKPYKCAICRRG

FLSSSSLHGHMQVHERNKDGSQSGSRMEDWKMKDTQKCSQCEEGFDFPEDLQKHIAECHPECSPNEDRAA

LQCVYCHELFVEETSLMNHMEQVHSGEKKNSCSICSESFHTVEELYSHMDSHQQPESCNHSNSPSLVTVG

YTSVSSTTPDSNLSVDSSTMVEAAPPIPKSRGRKRAAQQTPDMTGPSSKQAKVTYSCIYCNKQLFSSLAV

LQIHLKTMHLDKPEQAHICQYCLEVLPSLYNLNEHLKQVHEAQDPGLIVSAMPAIVYQCNFCSEVVNDLN

TLQEHIRCSHGFANPAAKDSNAFFCPHCYMGFLTDSSLEEHIRQVHCDLSGSRFGSPVLGTPKEPVVEVY

SCSYCTNSPIFNSVLKLNKHIKENHKNIPLALNYIHNGKKSRALSPLSPVAIEQTSLKMMQAVGGAPARP

AGEYICNQCGAKYTSLDSFQTHLKTHLDTVLPKLTCPQCNKEFPNQESLLKHVTIHFMITSTYYICESCD

KQFTSVDDLQKHLLDMHTFVFFRCTLCQEVFDSKVSIQLHLAVKHSNEKKVYRCTSCNWDFRNETDLQLH

VKHNHLENQGKVHKCIFCGESFGTEVELQCHITTHSKKYNCKFCSKAFHAIILLEKHLREKHCVFETKTP

NCGTNGASEQVQKEEVELQTLLTNSQESHNSHDGSEEDVDTSEPMYGCDICGAAYTMETLLQNHQLRDHN

IRPGESAIVKKKAELIKGNYKCNVCSRTFFSENGLREHMQTHLGPVKHYMCPICGERFPSLLTLTEHKVT

HSKSLDTGNCRICKMPLQSEEEFLEHCQMHPDLRNSLTGFRCVVCMQTVTSTLELKIHGTFHMQKTGNGS

AVQTTGRGQHVQKLYKCASCLKEFRSKQDLVKLDINGLPYGLCAGCVNLSKSASPGINVPPGTNRPGLGQ

NENLSAIEGKGKVGGLKTRCSSCNVKFESESELQNHIQTIHRELVPDSNSTQLKTPQVSPMPRISPSQSD

EKKTYQCIKCQMVFYNEWDIQVHVANHMIDEGLNHECKLCSQTFDSPAKLQCHLIEHSFEGMGGTFKCPV

CFTVFVQANKLQQHIFSAHGQEDKIYDCTQCPQKFFFQTELQNHTMTQHSS

>Cow521|772gi|119916162|ref|XP_582772.3| PREDICTED: hypothetical protein [Bos taurus]

MSRRKQAKPRSLKDPNCKLEDKTEDGEAIDCKKRPEDGEELEDEAVHSCDSCLQVFESLSDITEHKINQC

QLTDGVDVEDDPTCSWPASSPSSKDQTSPSHGEGCDFGEEEGGPGLPYPCQFCDKSFSRLSYLKHHEQSH

SDKLPFKCTYCSRLFKHKRSRDRHIKLHTGDKKYHCSECDAAFSRSDHLKIHLKTHTSNKPYKCAICRRG

FLSSSSLHGHMQVHERNKDGSQSGSRMEDWKMKDTQKCSQCEEGFDFPEDLQKHIAECHPECSPNEDRAA

LQCVYCHELFVEETSLMNHMEQMHGGEKKNSCSICSESFHSVEELYSHMDGHQQPESCNHSNSPSLVTVG

YTSVSSTTPDSNLSVDSSTMVEAAPPIPKSRGRKRAAQQTPDMTVPSSKQAKVTYSCIYCNKQLFSSLAV

LQIHLKTMHLDKPEQAHICQYCLEVLPSLYNLNEHLKQVHEAQDPGLIVSALPAIVYQCNFCSEVVNDLN

TLQEHIRCSHGFANPAAKDSNAFFCPHCYMGFLTDSSLEEHIRQVHCDLSGSRFGSPVLGTPKEPVVEVY

SCSYCTNSPIFNSVLKLNKHIKENHKNIPLALNYIHNGKKSRALSPLSPVAIEQTSLKMMQAVGGAPARQ

AGEYICNQCGAKYTSLDGFQTHLKTHLDTVLPKLTCPQCNKEFPNQESLLKHVTIHFMITSTYYICESCD

KQFTSVDDLQKHLLDMHTFVFFRCTLCQEVFDSKVSIQLHLAVKHSNEKKVYRCTSCNWDFRNETDLQLH

VKHNHLENQGKVHKCIFCGESFGTEVELQCHITTHSKKYNCQFCSKAFHAIILLEKHLREKHCVFDTKTP

NCGANGASEPGQKEEVELQTLLTNSQESHNSHDGSEEDVDTSEPMYGCDICGAAYTMETLLQNHQLRDHN

IRPGESAIVKKKAELIKGNYKCNVCSRTFFSENGLREHMQTHLGPVKHYMCPICGERFPSLLTLTEHKVT

HSKSLDTGNCRICKMPLQSEEEFLEHCQMHPDLRNSLTGFRCVVCMQTVTSTLELKIHGTFHMQKTGNGA

TVQATGRGPHVPKLYKCASCLKEFRSKQDLVKLDINGLPYGLCAGCVNLSKSASPGVNIAPGSSRPGLGQ

TENLGAVEGKGKASAPKTRCSSCNVKFESESELQNHIQTVHRELVPDSNSTQLKTPQVSPMPRISPSQSD

EKKTYQCIKCQMVFYNEWDIQVHVANHMIDEGLNHECKLCSQTFDSPAKLQCHLIEHSFEGMGGTFKCPV

CFTVFVQANKLQQHIFSAHGQEDKIYDCTQCPQKFFFQTELQNHTMTQHSS

>Dog521|633gi|73961326|ref|XP_547633.2| PREDICTED: similar to zinc finger protein 521 isoform 1 [Canis familiaris]

MSRRKQAKPRSLKDPNCKLEDKTEDGEAIDCKKRPEDGEELEDEAVHSCDSCLQVFESLSDITEHKINQC

QLTDGVDVEDDPTCSWPASSPSSKDQTSPSHGEGCDFGEEEGGPGLPYPCQFCDKSFSRLSYLKHHEQSH

SDKLPFKCTYCSRLFKHKRSRDRHIKLHTGDKKYHCSECDAAFSRSDHLKIHLKTHTSNKPYKCAICRRG

FLSSSSLHGHMQVHERNKDGSQSGSRMEDWKMKDTQKCSQCEEGFDFPEDLQKHIAECHPECSPNEDRAA

LQCIYCHELFVEETSLVNHMEQVHGGEKKNSCSICSETFHTVEELYSHMDSHQQPESCNHSNSPSLVTVG

YTSVSSTTPDSNLSVDSSTMVEAAPPIPKSRGRKRAAQQTPDMTVPSSKQAKVTYSCIYCNKQLFSSLAV

LQIHLKTMHLDKPEQAHICQYCLEVLPSLYNLNEHLKQVHEAQDPGLIVSAMPAIVYQCNFCSEVVNDLN

TLQEHIRCSHGFANPAAKDSNAFFCPHCYMGFLTDSSLEEHIRQVHCDLSGSRFGSPVLGTPKEPVVEVY

SCSYCTNSPIFNSVLKLNKHIKENHKNIPLALNYIHNGKKSRALSPLSPVAIEQTSLKMMQAVGGAPARP

AGEYICNQCGAKYTSLDSFQTHLKTHLDTVLPKLTCPQCNKEFPNQESLLKHVTIHFMITSTYYICESCD

KQFTSVDDLQKHLLDMHTFVFFRCTLCQEVFDSKVSIQLHLAVKHSNEKKVYRCTSCNWDFRNETDLQLH

VKHNHLENQGKVHKCIFCGESFGTEVELQCHITTHSKKYNCKFCSKAFHAIILLEKHLREKHCVFETKTP

NCGTNGASEQVQKEEVELQTLLTNSQESHNSHDGSEEDVDTSEPMYGCDICGAAYTMETLLQNHQLRDHN

IRPGESAIVKKKAELIKGNYKCNVCSRTFFSENGLREHMQTHLGPVKHYMCPICGERFPSLLTLTEHKVT

HSKSLDTGNCRICKMPLQSEEEFLEHCQMHPDLRNSLTGFRCVVCMQTVTSTLELKIHGTFHMQKTGNGS

TVQTTGRGQHVQKLYKCASCLKEFRSKQDLVKLDINGLPYGLCAGCVNLSKSGSPGINIPPGTNRPGLGQ

NENLSAIEGKGKAGGLKTRCSSCNVKFESESELQNHIQTVHRELVPDSNSTQLKTPQVSPMPRISPSQSD

EKKTYQCIKCQMVFYNEWDIQVHVANHMIDEGLNHECKLCSQTFDSPAKLQCHLIEHSFEGMGGTFKCPV

CFTVFVQANKLQQHIFSAHGQEDKIYDCTQCPQKFFFQTELQNHTMTQHSS

>Hu521|6276gi|24308069|ref|NP_056276.1| zinc finger protein 521 [Homo sapiens]

MSRRKQAKPRSLKDPNCKLEDKTEDGEALDCKKRPEDGEELEDEAVHSCDSCLQVFESLSDITEHKINQC

QLTDGVDVEDDPTCSWPASSPSSKDQTSPSHGEGCDFGEEEGGPGLPYPCQFCDKSFSRLSYLKHHEQSH

SDKLPFKCTYCSRLFKHKRSRDRHIKLHTGDKKYHCSECDAAFSRSDHLKIHLKTHTSNKPYKCAICRRG

FLSSSSLHGHMQVHERNKDGSQSGSRMEDWKMKDTQKCSQCEEGFDFPEDLQKHIAECHPECSPNEDRAA

LQCVYCHELFVEETSLMNHMEQVHSGEKKNSCSICSESFHTVEELYSHMDSHQQPESCNHSNSPSLVTVG

YTSVSSTTPDSNLSVDSSTMVEAAPPIPKSRGRKRAAQQTPDMTGPSSKQAKVTYSCIYCNKQLFSSLAV

LQIHLKTMHLDKPEQAHICQYCLEVLPSLYNLNEHLKQVHEAQDPGLIVSAMPAIVYQCNFCSEVVNDLN

TLQEHIRCSHGFANPAAKDSNAFFCPHCYMGFLTDSSLEEHIRQVHCDLSGSRFGSPVLGTPKEPVVEVY

SCSYCTNSPIFNSVLKLNKHIKENHKNIPLALNYIHNGKKSRALSPLSPVAIEQTSLKMMQAVGGAPARP

TGEYICNQCGAKYTSLDSFQTHLKTHLDTVLPKLTCPQCNKEFPNQESLLKHVTIHFMITSTYYICESCD

KQFTSVDDLQKHLLDMHTFVFFRCTLCQEVFDSKVSIQLHLAVKHSNEKKVYRCTSCNWDFRNETDLQLH

VKHNHLENQGKVHKCIFCGESFGTEVELQCHITTHSKKYNCKFCSKAFHAIILLEKHLREKHCVFETKTP

NCGTNGASEQVQKEEVELQTLLTNSQESHNSHDGSEEDVDTSEPMYGCDICGAAYTMETLLQNHQLRDHN

IRPGESAIVKKKAELIKGNYKCNVCSRTFFSENGLREHMQTHLGPVKHYMCPICGERFPSLLTLTEHKVT

HSKSLDTGNCRICKMPLQSEEEFLEHCQMHPDLRNSLTGFRCVVCMQTVTSTLELKIHGTFHMQKTGNGS

AVQTTGRGQHVQKLYKCASCLKEFRSKQDLVKLDINGLPYGLCAGCVNLSKSASPGINVPPGTNRPGLGQ

NENLSAIEGKGKVGGLKTRCSSCNVKFESESELQNHIQTIHRELVPDSNSTQLKTPQVSPMPRISPSQSD

EKKTYQCIKCQMVFYNEWDIQVHVANHMIDEGLNHECKLCSQTFDSPAKLQCHLIEHSFEGMGGTFKCPV

CFTVFVQANKLQQHIFSAHGQEDKIYDCTQCPQKFFFQTELQNHTMTQHSS

>Mu521|3467gi|21703968|ref|NP_663467.1| zinc finger protein 521 isoform 1 [Mus musculus]

MSRRKQAKPRSLKDPNCKLEDKIEDGEAVDCKKRPEDGEELEEDAVHSCDSCLQVFESLSDITEHKIHQC

QLTDGVDVEDDPSCSWPASSPSSKDQTSPSHGEGCDFGEEEGGPGLPYPCQFCDKSFSRLSYLKHHEQSH

SDKLPFKCTYCSRLFKHKRSRDRHIKLHTGDKKYHCSECDAAFSRSDHLKIHLKTHTSNKPYKCAVCRRG

FLSSSSLHGHMQVHERNKDGSQSGSRMEDWKMKDTQKCSQCEEGFDFPEDLQKHIAECHPECSPNEDRAA

LQCMYCHELFVEETSLMNHIEQVHGGEKKNSCSICSESFLTVEELYSHMDSHQQPESCNHSNSPSLVTVG

YTSVSSTTPDSNLSVDSSTMVEAAPPIPKSRGRKRAAQQTSDMTGPSSKQAKVTYSCIYCNKQLFSSLAV

LQIHLKTMHLDKPEQAHICQYCLEVLPSLYNLNEHLKQVHEAQDPGLIVSAMPAIVYQCNFCSEVVNDLN

TLQEHIRCSHGFANPAAKDSNAFFCPHCYMGFLTDSSLEEHIRQVHCDLSGSRFGSPVLGTPKEPVVEVY

SCSYCTNSPIFNSVLKLNKHIKENHKNIPLALNYIHNGKKSRALSPLSPVAIEQTTLKMMQTVGGGPARA

SGEYICNQCGAKYTSLDSFQTHLKTHLDTVLPKLTCPQCNKEFPNQESLLKHVTIHFMITSTYYICESCD

KQFTSVDDLQKHLLDMHTFVFFRCTLCQEVFDSKVSIQLHLAVKHSNEKKVYRCTSCNWDFRNETDLQLH

VKHNHLENQGKVHKCIFCGESFGTEVELQCHITTHSKKYNCRFCSKAFHAVILLEKHLREKHCVFETKTP

NCGTNGASEQVQKEEAELQTLLTNSQESHNSHDGSEEDVDSSEPMYGCDICGAAYTMETLLQNHQLRDHN

IRPGESAIVKKKAELIKGNYKCNVCSRTFFSENGLREHMQTHLGPVKHYMCPICGERFPSLLTLTEHKVT

HSKSLDTGNCRICKMPLQSEEEFLEHCQMHPDLRNSLTGFRCVVCMQTVTSTLELKIHGTFHMQKTGNGS

SVQTTGRGQHVQKLYKCASCLKEFRSKQDLVKLDINGLPYGLCAGCVNLSKSSSPGLSLPPGASRPGLGQ

NESLSAMEGKGKAGGLKTRCSSCNVKFESESELQNHIQTVHRELVPDANSTQLKTPQVSPMPRISPSQSD

EKKTYQCIKCQMVFYNEWDIQVHVANHMIDEGLNHECKLCSQTFDSPAKLQCHLIEHSFEGMGGTFKCPV

CFTVFVQANKLQQHIFSAHGQEDKIYDCTQCPQKFFFQTELQNHTMTQHSS

>Op521|4817gi|126321771|ref|XP_001364817.1| PREDICTED: hypothetical protein [Monodelphis domestica]

MTCDKIRHRDKETVNTQVVETLGALLIPMRPNCSSLQHQQHLYLEENEAEDHQEGVGQTATQSDPNCKLE

DKTEDGDALDCKKRPEDGEELEEEAVHSCDSCLQVFESLSDITEHKINQCQLTDGVDVEDDPTCSWPASS

PSSKDQTSPSHGEGCDFGEEEGGPGLPYPCQFCDKSFSRLSYLKHHEQSHSDKLPFKCTYCSRLFKHKRS

RDRHIKLHTGDKKYHCSECDAAFSRSDHLKIHLKTHTSNKPYKCAICRRGFLSSSSLHGHMQVHERNKDG

SQPGSRMEDWKMKDTQKCSQCEEGFDFPEDLQKHIAECHPECSPNEDRAALQCVYCHELFVEETSLLNHM

EQVHGGEKKNSCSICSESFHTVEELYSHMDSHQQPESCNHSNSPSLVTVGYTSVSSTTPDSNLSVDSSTM

VEAAPPLPKSRGRKRAAQQASDITGPSSKQAKVTYSCIYCNKQLFSSLAVLQIHLKTMHLDKPEQAHICQ

YCLEVLPSLYNLNEHLKQVHEAQDPALIVSAMPAMVYQCNFCSEVFNDLNTLQEHIRCSHGFANPAAKDS

NAFFCPHCYMGFLTDSSLEEHIRQVHCDLSSSRFGSPVLGTPKDPVVEVYSCSYCTNSPIFNSVLKLNKH

IKENHKNIPLALNYIHNGKKSRALSPLSPVTIEQTSLKMMQAVGGAPQRPTGEYICNQCGAKYTSLDSFQ

THLKTHLDTVLPKLTCPQCNKEFPNQESLLKHVTIHFMITSTYYICESCDKQFTSVDDLQKHLLDMHTFV

FFRCTLCQEVFDSKVSIQLHLAVKHSNEKKVYRCTSCNWDFRNETDLQLHVKHNHLENQGKVHKCIFCGE

SFGTEVELQCHITTHSKKYNCKFCSKAFHAIILLEKHLREKHCVFETKTPNCGTNGASEQVQKEEVELQT

LLTNSQESHNSHDGSEEDVDASEPMYGCDICGAAYTMETLLQNHQLRDHNIRPGESAIVKKKAELIKGNY

KCNVCSRTFFSENGLREHMQTHLGPVKHYMCPICGERFPSLLTLTEHKVTHSKSLDTGNCRICKMPLQSE

EEFLEHCQMHPDLRNSLTGFRCVVCMQTVTSTLELKIHGTFHMQKTGNGSAVQTTGRAQHLQKLYKCASC

LKEFRSKQDLVKLDINGLPYGLCAGCVNLSKSGSPNISIPSSSSRPGLGQNENLASMESKVKAGGLKTRC

SSCNVKFESESELQNHIQSIHRELVPDSNSTQLKTPQVSPMPRISPSQSDEKKTYQCIKCQMVFYNEWDI

QVHVANHMIDEGLNHECKLCNQTFDSPAKLQCHLIEHSFEGMGGTFKCPVCFTVFVQANKLQQHIFSAHG

QEDKIYDCTQCPQKFFFQTELQMDEMAVFCIICSPIHVMLCKSELEVDRCNLRGSLAHVYLRSSPVHTID

KAQRRVSQTGSSSKPDTGCRPDLVQKGLPVGSRSQESGRMMGRNDEVGLGGLYGPYQLRQTMMKPGIPAT

SRINSLQPWVG

>Rat521|019gi|109506968|ref|XP_001073019.1| PREDICTED: similar to zinc finger protein 521 isoform 1 [Rattus norvegicus]

MSRRKQAKPRSLKDPNCKLEDKIEDGEAVDCKKRPDDGEELEEDAVHSCDSCLQVFESLSDITEHKIHQC

QLTDGVDVEDDPTCSWPASSPSSKDQTSPSHGEGCDFGEEEGGPGLPYPCQFCDKSFSRLSYLKHHEQSH

SDKLPFKCTYCSRLFKHKRSRDRHIKLHTGDKKYHCSECDAAFSRSDHLKIHLKTHTSNKPYKCAVCRRG

FLSSSSLHGHMQVHERSKDGSQSGSRMEDWKMKDTQKCSQCEEGFDFPEDLQKHIAECHPECSPNEDRAA

LQCVYCHELFVEETSLMNHIEQVHGGEKKNSCSICSESFLTVEELYSHMDSHQQPESCNHSNSPSLVTVG

YTSVSSTTPDSNLSVDSSTMVEAAPPIPKSRGRKRAAQQTSDMTGPSSKQAKVTYSCIYCNKQLFSSLAV

LQIHLKTMHLDKPEQAHICQYCLEVLPSLYNLNEHLKQVHEAQDPGLIVSAMPAIIYQCNFCSEVVNDLN

TLQEHIRCSHGFANPAAKDSNAFFCPHCYMGFLTDSSLEEHIRQVHCDLSGSRFGSPVLGTPKEPVVEVY

SCSYCTNSPIFNSVLKLNKHIKENHKNIPLALNYIHNGKKSRALSPLSPVAIEQTSLKMMQTVGGGPARA

AGEYICNQCGAKYTSLDSFQTHLKTHLDTVLPKLTCPQCNKEFPNQESLLKHVTIHFMITSTYYICESCD

KQFTSVDDLQKHLLDMHTFVFFRCTLCQEVFDSKVSIQLHLAVKHSNEKKVYRCTSCNWDFRNETDLQLH

VKHNHLENQGKVHKCIFCGESFGTEVELQCHITTHSKKYNCRFCSKAFHAVLLLEKHLREKHCVFETKTP

NCGTNGASEQVQKEEAELQTLLTNSQESHNSHDGSEEDVDSSEPMYGCDICGAAYTMETLLQNHQLRDHN

IRPGESAIVKKKAELIKGNYKCNVCSRTFFSENGLREHMQTHLGPVKHYMCPICGERFPSLLTLTEHKVT

HSKSLDTGNCRICKMPLQSEEEFLEHCQMHPDLRNSLTGFRCVVCMQTVTSTLELKIHGTFHMQKTGNGS

SVQTTGRGQHVQKLYKCASCLKEFRSKQDLVKLDINGLPYGLCAGCVNLSKSSSPGLGLPPVASRPGLGQ

NESLSAMEGKGKAGGLKTTRCSSCNVKFESESELQNHIQTVHRELVPDANSTQLKTPQVSPMPRISPSQS

DEKKTYQCIKCQMVFYNEWDIQVHVANHMIDEGLNHECKLCSQTFDSPAKLQCHLIEHSFEGMGGTFKCP

VCFTVFVQANKLQQHIFSAHGQEDKIYDCTQCPQKFFFQTELQNHTMTQHSS

>Mac521|677gi|109121833|ref|XP_001097677.1| PREDICTED: similar to zinc finger protein 521 [Macaca mulatta]

MALEIIERNLNLEVQRRQKLHFAEFVVLTLSGVCKAPRGLWRWRHVILEKSVPSLGDCAPLYSRSTHCDI

PIAACSSSSSLAASTMDFLQDERAPECLQGALWPCPLGGGRRLLSLKGFTVSEMEELITENTLRTSSSKC

RGRRLQSGSWRAPSSLPLPALSPFNHAPLSVCECRQADNDFLSDYVQSESLRVRGPLWSHPDGFGGLASI

FHYYVDYRSDSAVQDLADPNCKLEDKTEDGEALDCKKRPEDGEELEDEAVHSCDSCLQVFESLSDITEHK

INQCQLTDGVDVEDDPTCSWPASSPSSKDQTSPSHGEGCDFGEEEGGPGLPYPCQFCDKSFSRLSYLKHH

EQSHSDKLPFKCTYCSRLFKHKRSRDRHIKLHTGDKKYHCSECDAAFSRSDHLKIHLKTHTSNKPYKCAI

CRRGFLSSSSLHGHMQVHERNKDGSQSGSRMEDWKMKDTQKCSQCEEGFDFPEDLQKHIAECHPECSPNE

DRAALQCVYCHELFVEETSLMNHMEQVHGGEKKNSCSICSESFHTVEELYSHMDSHQQPESCNHSNSPSL

VTVGYTSVSSTTPDSNLSVDSSTMVEAAPPIPKSRGRKRAAQQTPDMTGPSSKQAKVTYSCIYCNKQLFS

SLAVLQIHLKTMHLDKPEQAHICQYCLEVLPSLYNLNEHLKQVHEAQDPGLIVFAMPAIVYQCNFCSEVV

NDLNTLQEHIRCFHGFANPAAKDSNAFFCPHCYMGFFTDSSLEEHIRQVHCDLSGSRFGFPVLGTPKEPV

VEVYSCSYCTNSPIFNSVFKLNKHIKENHKNIPLALNYIHNGKKSRALSPLSPVAIEQTSLKMMQAVGGA

PARPAGEYICNQCGAKYTSLDSFQTHLKTHLDTVLPKLTCPQCNKEFPNQESLLKHVTIHFMITSTYYIC

ESCDKQFTSVDDLQKHLLDMHTFVFFRCTLCQEVFDSKVSIQLHLAVKHSNEKKVYRCTSCNWDFRNETD

LQLHVKHNHLENQGKVHKCIFCGESFGTEVELQCHITTHSKKYNCKFCSKAFHAIILLEKHLREKHCVFE

TKTPNCGTNGASEQVQKEEVELQTLLTNSQESHNSHDGSEEDVDTSEPMYGCDICGAAYTMETLLQNHQL

RDHNIRPGESAIVKKKAELIKGNYKCNVCSRTFFSENGLREHMQTHLGPVKHYMCPICGERFPSLLTLTE

HKVTHSKSLDTGNCRICKMPLQSEEEFLEHCQMHPDLRNSLTGFRCVVCMQTVTSTLELKIHGTFHMQKT

GNGSAVQTTGRGQHVQKLYKCASCLKEFRSKQDLVKLDINGLPYGLCAGCVNLSKSASPGINVPPGTNRP

GLGQNENLSAIEGKGKVGGLKTRCSSCNVKFESESELQNHIQTVHRELVPDSNSTQLKTPQVSPMPRISP

SQSDEKKTYQCIKCQMVFYNEWDIQVHVANHMIDEGLNHECKLCSQTFDSPAKLQCHLIEHSFEGMGGTF

KCPVCFTVFVQANKLQQHIFSAHGQEDKIYDCTQCPQKFFFQTELQNHTMTQHSS

>Xla521|4597gi|147905462|ref|NP_001084597.1| hypothetical protein LOC414550 [Xenopus laevis]

MSRRKQAKPRSLKDPNCKLEDTSEDGESPDCKKRQEEGDELEEEEAVHSCDSCLQVFESLSDITEHKISQ

CQLTDGADIEDDPTCSWPASSPSSKDQASPIHGEGFDFGEEEGIPGLPYPCQFCDKSFSRLSYLKHHEQS

HSDKLPFKCTYCSRLFKHKRSRDRHIKLHTGDKKYHCSECDASFSRSDHLKIHLKTHTSNKPYKCAICRR

GFLSSSSLHGHMQVHERNKDCSQSGSRMEEWKMKDTQKCSQCEEGFDFPEDLQKHIAECHPECSPNDDRG

ALQCMYCHELFMEETSLLNHMEQIHNSEKKNSCNICSENFHSVEELYSHMDSHQHPESCNPSNSPSLVTV

GYTSVSSTTPDSNLSVDSSTMVEVAPPLAKGRGRKRAVQQTGDAPTSKQARVSYSCIYCSKQLFSSLAVL

QIHLKTMHLDKPEQAHICQYCLEVLPSLFNLNEHLKQVHETQDPALIVSTMSAMVYQCNFCSEIFNDLNM

LQDHIRSSHGFPNPVTKDSNAFFCPHCYMGFLTDTSLEEHIRQVHCELGNSRFGSPVLGTPKEPVVEVYS

CSYCTNSPIFNSVLKLNKHIKENHKNIPLALNYIHNGKKSRALSPLSPITLEQSSLKMMQSLGGTPSRLA

GEYICNQCGAKYTSLDGFQTHLKTHLDTVLPKLTCPQCNKEFPNQESLLKHVTIHFMITSTYYICESCDK

QFTSVDDLQKHLLDMHTFGFFRCTLCQEVFDSKVSIQLHLAVKHSNEKKVYRCTSCNWDFRTETDLQLHV

KHNHLENQGKMHKCIFCGESFGTEVELQCHITTHSKKYNCKFCSKAFHAIILLEKHLREKHCVFEDKTQN

CGTNGASEQIQKEEVELQTLLTNNQESHNSHDGSEEDIDTSEPMYGCDICGAAYTMESLLQNHQLRDHNI

RPGESAIVKKKAELIKGNYKCNVCSRTFFSEGGLREHMQTHLGPVKHYMCPICGERFPSLLTLTEHKVTH

SKSLDTGNCRICKLPLQCEEDFLEHCQMHPDLRNSLTGFRCVVCMQTVTSTLELKIHGTFHMQKTGTASV

VQSAGRVQNLQKLYKCASCLKEFRSKQDLVKLDINGLPYGLCASCVNLSKSASPNANVTLSTNRQVISQT

DSLTCVEAKNYKTSVLKTRCSSCNVKFESETELQNHIQTIHRELTSENSATQLKTPQVSPMARISPQSDE

KKTYQCIKCQMVFYNEWDIQVHVANHMIDEGLNHECKLCSQTFDSPAKLQCHLIEHSFEGMGGTFKCPVC

FTVFVQANKLQQHIFSAHGQEDKIYDCAQCPQKFFFQTELQNHTMSQHSS

>Dan521|319gi|125843593|ref|XP_700319.2| PREDICTED: similar to Zinc finger protein 521, partial [Danio rerio]

MSRRKQAKPRALKDADAAAEIECKHEDEICLKSRISAEEEEEEEEEQLFEPVTAHKSHLTEDDPSCSWPA

SSPSSKDHTSPGHVDDYEYGEDEGGAGLPYPCQFCAKSFSRLSFLKCHEQSHRDKLPFSCTYCSRLFKHK

RSRDRHIKLHTGDKKYHCGDCDSAFSRSDHLKIHMKTHSANKPHKCPVCRRGFLSTNSLHGHMQVHERGK

DAANTRSPQEWRLKETRKCSRCEEGFDMPEELQKHIAESHPECSSPVLGSLEPGLQCIYCHEPFSNEGML

LSHIDQVHGRDRKSHVCTVCSEHFTTVEKLYAHMDIHQLPESSNSPSVLPIGYTSVSSTTPDSNLSVDSS

TMAETASIVPKTRGRRKRAAHNDMYRRSAKQPKIEYSCIYCSKQVFSSLGVLQIHLKTKHPDKPEQAHTC

QFCLEILPSLCNLNEHLKQIHNAEDPSGVLANLSEGLLQCNFCPELLGDLNALQEHIRCSHGFPNPVAKE

SNAFFCPKCFMGFLTEATLEEHVRQTHCDSGSMQFDSPLAVTPKDPVVEIYSCSYCTNSPIFNTVLKLNK

HIKENHKNIPLALNYINNGRRSLRVLSPSSPLSVEQGSLFKHNSSSLCSSEFICNQCGAKYTSLDLFQTH

LKTHLDGVVPQLTCPQCKKDFPNQESLLKHVAAHFTVMSTYYICESCDKQFTSVDDLQNHLLDMHTFVFF

RCTLCQEVFDSKVSTQLHLAVKHSNERKVYRCTSCTWDFKHEADLQMHVKHSHLENQGCSHRCIFCGESF

GTEVELQCHITTHSKKYNCHFCCKAFNAVYLLERHLRDKHCVFEGKAQNCSTNGSTSGSGDSVAKEEGDF

QGFLTNSHGGTGVGESHNSRDGSEEDFDSSEVMYSCDICGASYTMESLLTNHQLRDHNIRPGESAIHKRK

ADMIKGNHKCNVCSRTFFSEGGLREHMQTHLGPVKHYMCPICGERFPSLLTLTEHKVTHSKSLDTGSCRI

CKLPLQSEEDFLEHCQMHPDLRNSLTGFRCVVCMQTVTSTLELKIHGTFHMQKTGSANQPTACTPQLQSS

QKVVKCAACLKEFHSKHDLVKLDINGLTYGLCALCVTTGSSSSSSSSASPSISKTRCSSCNVKFESEAEL

QAHLQTVHRDQTPEAAQLRTPEGSPMSRKKTYQCIKCQMVFYSKWEIQVHVANHML

>Fugu521|Evi3 (ZNF521) lcl|Sequence 1 ORF:358..3549 Frame +1

MFCSYRWVLRYCTSVLHASLFPFLSSCPDGADLEDDPSCSWPASSPSSKDQTSPGHCEDYDFGEEEGGPG

LPYPCQFCDKSFSRLSFLKRHEQSHGDKLPFSCTFCSRLFKHKRSRDRHVKLHTGDKKYHCGECDSAFSR

SDHLKIHMKTHASNKPHKCPVCRRGFLSSSSLHGHMQVHERGKDGSASSLSRADEWKLKETRKCSRCEEG

FDVPEDLQRHIAECHPECSPSEDGGLGATLQCIYCHEPFSDEGTLLTHIDQAHSRDKKGHTCAICSEHFL

SVEDLYAHMDIHQLPESSNHSNSPSLLTVGYTSVSSTTPDSNLSVDSSTMVETAPPVPKTRGRRKRAAQN

ASDIGGRSSKQPKISYSCIYCNKQVFSSLAVLQIHLRTMHLDKPEQAHTCQYCLEVLPSLLNLNEHLKQV

HNAEDHAALLASLPDALLQCNFCPEVLSDLNALQEHIRCSHGFPSPVAKESNAFFCPQCFMGFLTETTLE

EHVRQTHCDGGSLRFDSPLAVTPKESIVEVYSCSYCTNSPIFNSVLKLNKHIKENHKNIPLALNYINNGK

KSLRTLSPSSPISVDQTAMLKQSSSRNVSEFICNQCGAKYTNLDLFQTHLKTHLDSLQPQLTCPQCNKEF

PNQESLLKHVTIHFTITSTYYICESCDKQFTSVDDLQKHLLDMHTFVFFRCTLCQEVFDSKVSTQLHLAV

KHSNEKKVYRCTSCNWDFRHETDLQLHVKHSHLENQGRAHRCIFCGESFGTEVELQCHITTHSKKYNCRF

CSKAFHAIVLLEKHLREKHCVFDGKPPNCGANGSTMGSVGDGHPKEDAELQGLMSNSHGPGAVGGSVLES

QNSHDGSEEEVDTAEPMFGCDICGASYTMESLLTNHQLRDHNIRPGESAMVKRKAEMIKGNHKCNVCSRT

FFSEAGLREHMQTHLGPVKHYMCPICGERFPSLLTLTEHKVTHSKSLDTGSCRICKMPLQCEEDFLEHCQ

MHPDLRNSLTGFRCVVCMQTVTSTLELKIHGTFHMQKTGMSANQPMGRGGVNCQNQQQHHIQKTFKCASC

LKDFRSKQDLVKLDINGLPYGLCASCVAAAGSKSSSPTINGGKQQQQGGAATPAVAPGAWVQGESLSPGE

GKGKAVSSSSSSSSTSSTSAAKTRCSSCNVKFESEAELHNHVQTVHREQAVDSNSGQLKTPQVSPMPRAS

PSQTEEVERALLYSRSC

>Stickle521|ZNF521 lcl|Sequence 1 ORF:23293..26472 Frame +1

MKTHASNKPHKCPVCRRGFLSSSSLHGHMQVHERGKDGNSSSLSRADEWKLKETRKCSRCEEGFDVPEEL

QRHIAECHPECSPSEDGGLGATLQCIYCHEPFGDEGTLLTHIDQAHGRDRKGHSCAICSEHFLSVEDLYA

HMDVHQLPESSNHSNSPSLLTVGYTSVSSTTPDSNLSVDSSTMVETAPPVPKTRGRRKRAAQNTSDMGGR

SSKQPKVSYSCIYCNKQVFSSLAVLQIHLRTMHLDKPEQAHTCQFCLEVLPSLLNLNEHLKQVHNAEDHA

ALLASLPDALLQCNFCPEVLSDLNGLQEHIRCSHGFPSPVAKESNAFFCPQCFMGFLTETTLEEHVRQTH

CDGGSLRFDSPLAVTPKESIVEVYSCSYCTNSPIFNSVLKLNKHIKENHKNIPLALNYINNGKKSLRTLS

PSSPISVEHTLLKQGGSAPRSAGEFICNQCGAKYTSLDLFQTHLKTHLDGMQPQLTCPQCNKEFPNQESL

LKHVTLHFTITSTYYICESCDKQFTSVDDLQKHLLDMHTFVFFRCTLCQEVFDSKVSTQLHLAVKHSNEK

KVYRCTSCNWDFRHETDLQLHVKHSHLENQGRAHRCIFCGESFGTEVELQCHITTHSKKYNCRFCSKAFH

AIVLLEKHLREKHCVFEGKAQNCGANGSTGSGVDGQPKEDVELHGLLTNSHGSGVAAGTVVESQNSHDAS

EEEVDTADPMYGCDICGASYTMDSLLTNHQLRDHNIRPGESAMMKRKADMIKGNHKCNVCSRTFFSEPGL

REHMQTHLGPVKHYMCPICGERFPSLLTLTEHKVTHSKSLDTGSCRICKMPLHSEEDFLEHCQMHPDLRN

SLTGFRCVVCMQTVTSTLELKIHGTFHMQKTGTMSGNHQPMGRSHVISHNQQQHHIQKPFKCASCLKDFR

SKQDLVKLDINGLPYGLCASCVTAAGSKSSSPTVNGGRQQQQHGGAITPATTTAAWIQGESLSPGDVKGK

AVSSSSSSCSTSSVSAAKTRCSSCNVKFESEAELQNHVQTVHREQAGDSNGGQLKTPQVSPMPRSSPSQT

EEVLYFGLA

>Gal423|103gi|118096326|ref|XP_414103.2| PREDICTED: similar to zinc finger protein 423 [Gallus gallus]

MAVEEGESSDFALNWDSSVTQSGGLGGELESDAKDSRALEERNSVTSQEERNEEDEDMEDESIYTCDNCQ

QDFDSLADLTEHRAHHCPGDGDDDPQLSWVASSPSSKDVASPTQMIGDGCDLGIGEEEGGTGLPYPCQFC

DKSFIRLSYLKRHEQIHSDKLPFKCTYCSRLFKHKRSRDRHIKLHTGDKKYHCHECEAAFSRSDHLKIHL

KTHSSSKPFKCTVCKRGFSSTSSLQSHMQAHKKNKEHMAKSEKEVKKDDFMCDYCEETFSQTEELEKHVM

TRHPQLSEKADLQCIHCPEVFSDENLLLSHIHQAHANKKHKCPMCPEQFSSVEEVYCHLDSHRQPDSSNH

SISPDPVLGSVASMSSATPDSSASVERGSTPDSTLKPLRGQKKIRSVDREEGQNWSKVTYSCPYCSKRDF

NSLAVLEIHLKTIHADKPQQSHTCQICLDSMPTLYNLNEHVRKVHKNHAYPMMQFSNISAFHCNYCPEMF

ADINSLQEHIRITHCGPNATPQDGNNAFFCNQCSMGFLTEATLTEHIQQTHCNVGNSKLDSPVIQPTQSF

MEVYSCPYCTNSPIFGSILKLTKHIKENHKNIPLAHNKKSKAEQSPVSSDVEVSSPKRQRLSASVNSVSN

GEYPCNQCDLKFSNFESFQTHLKLHLELLLRKQSCPQCKEDFDSQESLLQHLTVHYMTTSTHYVCESCDK

QFSSVDDLQKHLLDMHTFVLYHCTLCQEVFDSKVSIQVHLAVKHSNEKKMYRCTACNWDFRKEVDLQIHV

KHSHLGNPSKSHKCIFCGETFSTEVELQCHITTHSKKYNCKFCSKAFHAIILLEKHLREKHCVFDASAEN

GTANGMTPTNKKTEGADIQNMLMKNPDTSNSHEASEDDVDASEPMYGCDICGAAYTMEVLLQNHRLRDHN

IRPGEDDCSRKKAEFIKGSHKCNICSRTFFSENGLREHMQTHRGPAKHYMCPICGERFPSLLTLTEHKVT

HSKSLDTGTCRICKMPLQSEEEFIEHCQMHPDLRNSLTGFRCVVCMQTVTSTLELKIHGTFHMQKLAGNS

ATSSPNGQALQKLYKCALCLKEFRNKQDLVKLDVNGLPYGLCAGCMTRSTNGQSSGLTPQEVNERPCGSL

RCPECSVKFESAEDLESHIQVDHRDLTPETSGQRKGTQTSPVPRKKTYQCIKCQMTFENEREIQIHVANH

MIEEGINHECKLCNQMFDSPAKLLCHLIEHSFEGMGGTFKCPVCFTVFVQANKLQQHIFAVHGQEDKIYD

CSQCPQKFFFQTELQNHTLSQHAQ

>Pan423|978gi|114662385|ref|XP_001163978.1| PREDICTED: zinc finger protein 423 [Pan troglodytes]

MSRRKQAKPRSVKVEEGEASDFSLAWDSSVTAAGGLEGEPECDQKTSRALEDRNSVTSQEERNEDDEDME

DESIYTCDHCQQDFESLADLTDHRAHRCPGDGDDDPQLSWVASSPSSKDVASPTQMIGDGCDLGLGEEEG

GTGLPYPCQFCDKSFIRLSYLKRHEQIHSDKLPFKCTYCSRLFKHKRSRDRHIKLHTGDKKYHCHECEAA

FSRSDHLKIHLKTHSSSKPFKCTVCKRGFSSTSSLQSHMQAHKKNKEHLAKSEKEAKKDDFMCDYCEDTF

SQTEELEKHVLTRHPQLSEKADLQCIHCPEVFVDENTLLAHIHQAHANQKHKCPMCPEQFSSVEGVYCHL

DSHRQPDSSNHSVSPDPVLGSVASMSSATPDSSASVERGSTPDSTLKPLRGQKKMRDDGQGWTKVVYSCP

YCSKRDFNSLAVLEIHLKTIHADKPQQSHTCQICLDSMPTLYNLNEHVRKLHKNHAYPVMQFGNISAFHC

NYCPEMFADINSLQEHIRVSHCGPNANPSDGNNAFFCNQCSMGFLTESSLTEHIQQAHCSVGSAKLESPV

VQPTQSFMEVYSCPYCTNSPIFGSILKLTKHIKENHKNIPLAHSKKSKAEQSPVSSDVEVSSPKRQRLSA

SANSISNGEYPCNQCDLKFSNFESFQTHLKLHLELLLRKQACPQCKEDFDSQESLLQHLTVHYMTTSTHY

VCESCDKQFSSVDDLQKHLLDMHTFVLYHCTLCQEVFDSKVSIQVHLAVKHSNEKKMYRCTACNWDFRKE

ADLQVHVKHSHLGNPAKAHKCIFCGETFSTEVELQCHITTHSKKYNCKFCSKAFHAIILLEKHLREKHCV

FDAATENGTANGVPPTATKKAEPADLQGMLLKNPEAPNSHEASEDDVDASEPMYGCDICGAAYTMEVLLQ

NHRLRDHNIRPGEDDGSRKKAEFIKGSHKCNVCSRTFFSENGLREHLQTHRGPAKHYMCPICGERFPSLL

TLTEHKVTHSKSLDTGTCRICKMPLQSEEEFIEHCQMHPDLRNSLTGFRCVVCMQTVTSTLELKIHGTFH

MQKLAGSSAASSPNGQGLQKLYKCALCLKEFRSKQDLVKLDVNGLPYGLCAGCMARSANGQVGGLAPPEP

ADRPCAGLRCPECSVKFESAEDLESHMQVDHRDLTPETSGPRKGTQTSPVPRKKTYQCIKCQMTFENERE

IQIHVANHMIEEGINHECKLCNQMFDSPAKLLCHLIEHSFEGMGGTFKCPVCFTVFVQANKLQQHIFAVH

GQEDKIYDCSQCPQKFFFQTELQNHTMSQHAQ

>Cow423|109gi|119910017|ref|XP_876109.2| PREDICTED: similar to zinc finger protein 423 [Bos taurus]

MLTEEEVGVRLTLLMSLSEEVFASCSFFLGSPSEDFFELYEEFSQSGPRDLDPIEHNQVERVFSVLVDAF

KPYSEMTAAQFSVPVPAPGPYCQPREMLQGPGALVVALKAVRRSGGHGVRQWMLRGARPWLAKPGVCSSS

AALLCTEQPSESPGPAPASSGPDVFFRMGSLPASGPGGCRPPASSYIPCRLPGPFWGLAFGGAPCCDFLR

LVAPRLSQVLALGSGPRLHSTGRFHAPFVWDVVTAPLCMAERALGSVTFHQSWTAVEEGEASDFSLAWDS

SVTASGGLEGEPECDRKTSRVLEDRNSVTSQEERNEDDEDVEDESIYTCDHCQQDFESLAELTDHRAHRC

PGDGDDDPQLSWVASSPSSKDVASPTQMIGDGCDLGLGEEEGGTGLPYPCQFCDKSFIRLSYLKRHEQIH

SDKLPFKCTYCSRLFKHKRSRDRHIKLHTGDKKYHCHECEAAFSRSDHLKIHLKTHSSSKPFKCTVCKRG

FSSTSSLQSHMQAHKKNKEHLAKSEKEAKKDDFMCDYCEDTFSQTEELEKHVLTRHPQLSEKADLQCIHC

PEVFVDENALLAHIHQAHANQKHKCPMCPEQFSSVEGVYCHLDSHRQPDSSNHSVSPDPVLGSVASMSSA

TPDSSASVERGSTPDSTLKPLRGQKKMRDDGQGWSKVVYSCPYCSKRDFNSLAVLEIHLKTIHADKPQQS

HTCQICLDSMPTLYNLNEHVRKLHKNHAYPVMQFGSISAFHCNYCPEMFADINSLQEHIRVSHCGPNANP

PDGNNAFFCNQCSMGFLTESSLTEHIQQAHCSVGSAKLESPVVQPTQSFMEVYSCPYCTNSPIFGSILKL

TKHIKENHKNIPLAHSKKSKAEQSPVSSDVEVSSPKRQRLSASANSISNGEYPCNQCDLKFSNFESFQTH

LKLHLELLLRKQACPQCKEDFDSQESLLQHLTVHYMTTSTHYVCESCDKQFSSVDDLQKHLLDMHTFVLY

HCTLCQEVFDSKVSIQVHLAVKHSNEKKMFRCTACNWDFRKEADLQVHVKHSHLGNPARAHKCIFCGETF

STEVELQCHITTHSKKYNCKFCSKAFHAIILLEKHLREKHCVFDAAAENGTANGVPPAAAKKAEPADLPG

VLLKNPEAPNSHEASEDDVDASEPMYGCDICGAAYTMEVLLQNHRLRDHNIRPGEDDGSRKKAEFIKGSH

KCNVCSRTFFSENGLREHLQTHRGPAKHYMCPICGERFPSLLTLTEHKVTHSKSLDTGTCRICKMPLQSE

EEFIEHCQMHPDLRNSLTGFRCVVCMQTVTSTLELKIHGTFHMQKLAGSSAASSPNGQGLQKLYKCALCL

KEFRSKQDLVKLDVNGLPYGLCAGCMARSANGQVGGLAPPEPADRPCAGLRCPECSVKFESAEDLESHMQ

VDHRDLTPETSGPRKGAQTSPVPRKKTYQCIKCQMTFENEREIQIHVANHMIAQRMASLLLLLRHFK

>Dog423|417gi|73950414|ref|XP_544417.2| PREDICTED: similar to zinc finger protein 423 [Canis familiaris]

MHLATSSDVFLGYFTATQSKFLGSQANASWVPAPFFCDSEPAAGGEGERPIPEELASQKPPAHFPHLGSL

KDRTMRGFPETLLAPVQWEVPGLEDFEEKAAKPWERGLEGEPECDRKTSRALEDRNSVTSQEERNEDDED

MEDESIYTCDHCQQDFECLADLTDHRAHRCPGDGDDDPQLSWVASSPSSKDVASPTQMIGDGCDLGLGEE

EGGTGLPYPCQFCDKSFIRLSYLKRHEQIHSDKLPFKCTYCSRLFKHKRSRDRHIKLHTGDKKYHCHECE

AAFSRSDHLKIHLKTHSSSKPFKCTVCKRGFSSTSSLQSHMQAHKKNKEHLAKSEKEAKKDDFMCDYCED

TFSQTEELEKHVLTRHPQLSEKADLQCIHCPEVFVDENALLAHIHQAHANQKHKCPMCPEQFSSVEGVYC

HLDSHRQPDSSNHSVSPDPVLGSVASMSSATPDSSASVERGSTPDSTLKPLRGPKKLRDDGQGWSKVVYS

CPYCSKRDFHSLAVLEIHLKTIHADKPQQSHTCQICLDSMPTLYNLNEHVRKLHKNHAYPVMQFGNISAF

HCNYCPEMFADINSLQEHIRVSHCGPNVNPPDGNNAFFCNQCSMGFLTESSLTEHIQQAHCGVGGAKLES

PVVQPAQSFMEVYSCPYCTNSPIFGSILKLTKHIKENHKNIPLAHSKKSKAEQSPVSSDVEVSSPKRQRL

SASANSISNGEYPCNQCDLKFSNFESFQTHLKLHLELLLRKQACPQCKEDFDSQESLLQHLTVHYMTTST

HYVCESCDKQFSSVDDLQKHLLDMHTFVLYHCTLCQEVFDSKVSIQVHLAVKHSNEKKMYRCTACNWDFR

KEADLQVHVKHSHLGNPAKAHKCIFCGETFSTEVELQCHITTHSKKYNCKFCSKAFHAIILLEKHLREKH

CVFDAATENGTANGVPPTAAAAAAAKKTEPADLPGMLLKNPEAPNSHEASEDDVDASEPMYGCDICGAAY

TMEVLLQNHRLRDHNIRPGEDDGSRKKAEFIKGSHKCNVCSRTSSRRTGSGSTCRRTGAPPSTTCAPSAA

SASLPC

>Human423|EBFAZ protein from exons 1,2,5,6 etc zinc finger protein 423 [Homo sapiens]

MSRRKQAKPRSVKVEEGEASDFSLAWDSSVTAAGGLEGEPECDQKTSRALEDRNSVTSQEERNEDDEDME

DESIYTCDHCQQDFESLADLTDHRAHRCPGDGDDDPQLSWVASSPSSKDVASPTQMIGDGCDLGLGEEEG

GTGLPYPCQFCDKSFIRLSYLKRHEQIHSDKLPFKCTYCSRLFKHKRSRDRHIKLHTGDKKYHCHECEAA

FSRSDHLKIHLKTHSSSKPFKCTVCKRGFSSTSSLQSHMQAHKKNKEHLAKSEKEAKKDDFMCDYCEDTF

SQTEELEKHVLTRHPQLSEKADLQCIHCPEVFVDENTLLAHIHQAHANQKHKCPMCPEQFSSVEGVYCHL

DSHRQPDSSNHSVSPDPVLGSVASMSSATPDSSASVERGSTPDSTLKPLRGQKKMRDDGQGWTKVVYSCP

YCSKRDFNSLAVLEIHLKTIHADKPQQSHTCQICLDSMPTLYNLNEHVRKLHKNHAYPVMQFGNISAFHC

NYCPEMFADINSLQEHIRVSHCGPNANPSDGNNAFFCNQCSMGFLTESSLTEHIQQAHCSVGSAKLESPV

VQPTQSFMEVYSCPYCTNSPIFGSILKLTKHIKENHKNIPLAHSKKSKAEQSPVSSDVEVSSPKRQRLSA

SANSISNGEYPCNQCDLKFSNFESFQTHLKLHLELLLRKQACPQCKEDFDSQESLLQHLTVHYMTTSTHY

VCESCDKQFSSVDDLQKHLLDMHTFVLYHCTLCQEVFDSKVSIQVHLAVKHSNEKKMYRCTACNWDFRKE

ADLQVHVKHSHLGNPAKAHKCIFCGETFSTEVELQCHITTHSKKYNCKFCSKAFHAIILLEKHLREKHCV

FDAATENGTANGVPPMATKKAEPADLQGMLLKNPEAPNSHEASEDDVDASEPMYGCDICGAAYTMEVLLQ

NHRLRDHNIRPGEDDGSRKKAEFIKGSHKCNVCSRTFFSENGLREHLQTHRGPAKHYMCPICGERFPSLL

TLTEHKVTHSKSLDTGTCRICKMPLQSEEEFIEHCQMHPDLRNSLTGFRCVVCMQTVTSTLELKIHGTFH

MQKLAGSSAASSPNGQGLQKLYKCALCLKEFRSKQDLVKLDVNGLPYGLCAGCMARSANGQVGGLAPPEP

ADRPCAGLRCPECSVKFESAEDLESHMQVDHRDLTPETSGPRKGTQTSPVPRKKTYQCIKCQMTFENERE

IQIHVANHMIEEGINHECKLCNQMFDSPAKLLCHLIEHSFEGMGGTFKCPVCFTVFVQANKLQQHIFAVH

GQEDKIYDCSQCPQKFFFQTELQNHTMSQHAQ

>Mu423|1584gi|46359077|ref|NP_201584.2| zinc finger protein 423 [Mus musculus]

MSRRKQAKPRSVKVEEGEASDFSLAWDSSVAAAGGLEGEPECDRKTSRALEDRNSVTSQEERNEDDEDVE

DESIYTCDHCQQDFESLADLTDHRAHRCPGDGDDDPQLSWVASSPSSKDVASPTQMIGDGCDLGLGEEEG

GTGLPYPCQFCDKSFIRLSYLKRHEQIHSDKLPFKCTFCSRLFKHKRSRDRHIKLHTGDKKYHCHECEAA

FSRSDHLKIHLKTHSSSKPFKCSVCKRGFSSTSSLQSHMQAHKKNKEHLAKSEKEAKKDDFMCDYCEDTF

SQTEELEKHVLTLHPQLSEKADLQCIHCPEVFVDESTLLAHIHQAHANQKHKCPMCPEQFSSVEGVYCHL

DSHRQPDSSNHSVSPDPVLGSVASMSSATPDSSASVERGSTPDSTLKPLRGQKKMRDDGQSWPKVVYSCP

YCSKRDFTSLAVLEIHLKTIHADKPQQSHTCQICLDSMPTLYNLNEHVRKLHKSHAYPVMQFGNISAFHC

NYCPEMFADINSLQEHIRVSHCGPNANPPDGNNAFFCNQCSMGFLTESSLTEHIQQAHCSVGSTKLESPV

VQPTQSFMEVYSCPYCTNSPIFGSILKLTKHIKENHKNIPLAHSKKSKAEQSPVSSDVEVSSPKRQRLSG

SANSISNGEYPCNQCDLKFSNFESFQTHLKLHLELLLRKQACPQCKEDFDSQESLLQHLTVHYMTTSTHY

VCESCDKQFSSVDDLQKHLLDMHTFVLYHCTLCQEVFDSKVSIQVHLAVKHSNEKKMYRCTACNWDFRKE

ADLQVHVKHSHLGNPAKAHKCIFCGETFSTEVELQCHITTHSKKYNCRFCSKAFHAVILLEKHLREKHCV

FDAAAENGTANGVPPTSTKKAEPADLQGMLLKNPEAPNSHEASEDDVDASEPMYGCDICGAAYTMEVLLQ

NHRLRDHNIRPGEDDGSRKKAEFIKGSHKCNVCSRTFFSENGLREHLQTHRGPAKHYMCPICGERFPSLL

TLTEHKVTHSKSLDTGTCRICKMPLQSEEEFIEHCQMHPDLRNSLTGFRCVVCMQTVTSTLELKIHGTFH

MQKLAGSSAASSPNGQGLQKLYKCALCLKEFRSKQDLVRLDVNGLPYGLCAGCMARSANGQVGGLAPPEP

ADRPCAGLRCPECNVKFESAEDLESHMQVDHRDLTPETSGPRKGAQTSPVPRKKTYQCIKCQMTFENERE

IQIHVANHMIEEGINHECKLCNQMFDSPAKLLCHLIEHSFEGMGGTFKCPVCFTVFVQANKLQQHIFAVH

GQEDKIYDCSQCPQKFFFQTELQNHTMSQHAQ

>Op423|1030gi|126296289|ref|XP_001371030.1| PREDICTED: similar to zinc finger protein 423 [Monodelphis domestica]

MYYGFGGGLGGEQENDTKGSRALEDRNSVTSQEERNEEDEDMEDESIYTCDNCQQDFESLADLTDHRAHR

CPGDGDDDPQLSWVASSPSSKDVASPTQMIGDGCDLGIGEEEGGTGLPYPCQFCDKSFIRLSYLKRHEQI

HSDKLPFKCTYCSRLFKHKRSRDRHIKLHTGDKKYHCHECEAAFSRSDHLKIHLKTHSSSKPFKCTVCKR

GFSSTSSLQSHMQAHKKNKEHVAKSEKETKKDDFMCDYCEDTFSQTEELEKHVLTRHPQLSEKADLQCIH

CPEVFVDENSLLAHIHQAHANKKHKCPMCPEQFSSVEEVYCHLDSHRQPDSSNHSISPDPVLGSVASMSS

ATPDSSASVERGSTPDSTLKPLRGQKKIRAVDREEGQSWSKVTYSCPYCSKRDFNSLAVLEIHLKTIHVD

KPQQSHTCQVCLDSMPTLYNLNEHVRKVHKNHAYPMMQFSNISAFHCNYCPEMFADINSLQEHIRVTHCG

PNANPADGNNAFFCNQCSMGFLTESSLTEHIQQTHCNVGNSKLESPVVQPTQSFMEVYSCPYCTNSPIFG

SILKLTKHIKENHKNIPLAHNKKSKAEQSPVSSDVEVSSPKRQRLSASINSVSNGEYPCNQCDLKFSNFE

SFQTHLKLHLELLLRKQSCPQCKEDFDSQDSLLQHLTVHYMTTSTHYVCESCDKQFSSVDDLQKHLLDMH

TFVLYHCTLCQEVFDSKVSIQVHLAVKHSNEKKMYRCTACNWDFRKEVDLQIHVKHSHLGNPTKSHKCIF

CGETFSTEVELQCHITTHSKKYNCKFCSKAFHAIILLEKHLREKHCVFDAAAENGTANGMPPASKKAEPA

DIQNMLMKNPEAPNSHEASEDDVDASEPMYGCDICGAAYTMEVLLQNHRLRDHNIRPGEDDCSRKKAEFI

KGSHKCNVCSRTFFSENGLREHMQTHRGPAKHYMCPICGERFPSLLTLTEHKVTHSKSLDTGTCRICKMP

LQSEEEFIEHCQMHPDLRNSLTGFRCVVCMQTVTSTLELKIHGTFHMQKLAGNSAASSPNGQSLQKLYKC

ALCLKEFRNKQDLVKLDVNGLPYGLCAGCMTRSTNGQVSGMTPPEPSERPCGSLRCPECSVKFESAEDLE

SHVQMDHRDLTPETSGQRKGAQTSPVPRKKTYQCIKCQMTFENEREIQIHVANHMIEEGINHECKLCNQM

FDSPAKLLCHLIEHSFEGMGGTFKCPVCFTVFVQANKLQQHIFAVHGQEDKIYDCSQCPQKFFFQTELQI

SSSFLLVSLAALGLQELETFVQGLMNGRERAASPLMPLPKAKIQEVVGVRRGLSCLGWMPPLAASMEAGS

FSALVGLGYSLYCSTTHAVSQCVAVALQELE

>Rat423|035gi|16758356|ref|NP_446035.1| zinc finger protein 423 [Rattus norvegicus]

MIGDGCDLGLGEEEGGTGLPYPCQFCDKSFIRLSYLKRHEQIHSDKLPFKCTFCSRLFKHKRSRDRHIKL

HTGDKKYHCHECEAAFSRRDHLKIHLKTHSSSKPFKCSVCKRGFSSTSSLQSHMQAHKKNKEHLAKSEKE

AKKDDFMCDYCEDTFSQTEELEKHVLTLHPQLSEKADLQCIHCPEVFVDESTLLAHIHQAHANQKHKCPM

CPEQFSSVEGVYCHLDSHRQPDSSNHSVSPDPVLGSVASMSSATPDSTPDPVLGSVASMSSATPDSSASV

ERGSTPDSTLKPLRGQKKMRDDGQSWSKVVYSCPYCSKRDFTSLAVLEIHLKTIHADKPQQSHTCQICLD

SMPTLYNLNEHVRKLHKSHAYPVMQFGNISAFHCNYCPEMFADINSLQEHIRVSHCGPNANPPDGNNAFF

CNQCSMGFLTESSLTEHIQQAHCSVGSTKLESPVIQPTQSFMEVYSCPYCTNSPIFGSILKLTKHIKENH

KNIPLAHSKKSKAEQSPVSSDVEVSSPKRQRLSGSANSISNGEYPCNQCDLKFSNFESFQTHLKLHLELL

LRKQACPQCKEDFDSQESLLQHLTVHYMTTSTHYVCESCDKQFSSVDDLQKHLLDMHTFVLYHCTLCQEV

FDSKVSIQVHLAVKHSNEKKMYRCTACNWDFRKEADLQVHVKHSHLGNPAKAHKCIFCGETFSTEVELQC

HITTHSKKYNCRFCSKAFHAVLLLEKHLREKHCVFDPAAENGTANGVPPTSTKKAEPADLQGMLLKNPEA

PNSHEASEDDVDASEPMYGCDICGAAYTMEVLLQNHRLRDHNIRPGEDDGSRKKAEFIKGSHKCNVCSRT

FFSENGLREHLQTHRGPAKHYMCPICGERFPSLLTLTEHKVTHSKSLDTGTCRICKMPLQSEEEFIEHCQ

MHPDLRNSLTGFRCVVCMQTVTSTLELKIHGTFHMQKLAGSSAASSPNGQGLQKLYKCALCLKEFRSKQD

LVRLDVNGLPYGLCAGCMARSANGQVGGLAPPEPADRPCAGLRCPECNVKFESAEDLESHMQVDHRDLTP

ETSGPRKGAQTSPVPRKKTYQCIKCQMTFENEREIQIHVANHMIEEGINHECKLCNQMFDSPAKLLCHLI

EHSFEGMGGTFKCPVCFTVFVQANKLQQHIFAVHGQEDKIYDCSQCPQKFFFQTELQNHTMSQHAQ

>Mac423|507gi|109128453|ref|XP_001082507.1| PREDICTED: similar to zinc finger protein 423 isoform 1 [Macaca mulatta]

MSRRKQAKPRSVKVEEGEASDFSLAWDSSVTAAGGLEGEPECDRKTSRALEDRNSVTSQEERNEDDEDME

DESIYTCDHCQQDFESLADLTDHRAHRCPGDGDDDPQLSWVASSPSSKDVASPTQMIGDGCDLGLGEEEG

GTGLPYPCQFCDKSFIRLSYLKRHEQIHSDKLPFKCTYCSRLFKHKRSRDRHIKLHTGDKKYHCHECEAA

FSRSDHLKIHLKTHSSSKPFKCTVCKRGFSSTSSLQSHMQAHKKNKEHLAKSEKEAKKDDFMCDYCEDTF

SQTEELEKHVLTRHPQLSEKADLQCIHCPEVFVDENTLLTHIHQAHANQKHKCPMCPEQFSSVEGVYCHL

DSHRQPDSSNHSVSPDPVLGSVASMSSATPDSSASVERGSTPDSTLKPLRGQKKMRDDGQGWTKVVYSCP

YCSKRDFNSLAVLEIHLKTIHADKPQQSHTCQICLDSMPTLYNLNEHVRKLHKNHAYPMMQFGNISAFHC

NYCPEMFADINSLQEHIRVSHCGPNTNPSDGNNAFFCNQCSMGFLTESSLTEHIQQAHCSVGSAKLESPV

VQPTQSFMEVYSCPYCTNSPIFGSILKLTKHIKENHKNIPLAHSKKSKAEQSPVSSDVEVSSPKRQRLSA

SANSISNGEYPCNQCDLKFSNFESFQTHLKLHLELLLRKQACPQCKEDFDSQESLLQHLTVHYMTTSTHY

VCESCDKQFSSVDDLQKHLLDMHTFVLYHCTLCQEVFDSKVSIQVHLAVKHSNEKKMYRCTACNWDFRKE

ADLQVHVKHSHLGNPAKAHKCIFCGETFSTEVELQCHITTHSKKYNCKFCSKAFHAIILLEKHLREKHCV

FDAATENGTANGVPPTATKKAEPADLQGMLLKNPEAPNSHEASEDDVDASEPMYGCDICGAAYTMEVLLQ

NHRLRDHNIRPGEDDGSRKKAEFIKGSHKCNVCSRTFFSENGLREHLQTHRGPAKHYMCPICGERFPSLL

TLTEHKVTHSKSLDTGTCRICKMPLQSEEEFIEHCQMHPDLRNSLTGFRCVVCMQTVTSTLELKIHGTFH

MQKLAGSSAASSPNGQGLQKLYKCALCLKEFRSKQDLVKLDVNGLPYGLCAGCMARSANGQVGGLVPPEP

ADRPCAGLRCPECSVKFESAEDLESHMQVDHRDLTPETSGPRKATQTSPVPRVSPSRSQEQTAHRKTRCH

QLFFLDKDINVQVGGHSLEEGINHECKLCNQMFDSPAKLLCHLIEHSFEGMGGTFKCPVCFTVFVQANKL

QQHIFAVHGQEDKIYDCSQCPQKFFFQTELQNHTMSQHAQ

>Dan423|499gi|121583818|ref|NP_001073499.1| similar to mKIAA0760 protein [Danio rerio]

MSRRKQAKPRSVKAVEEAESTECASGWDSSVQTDAAVSERDSDRKESRAVGEDGEQSVTSHDERVGEEDL

DDDSIFTCDNCQQDFECLADLTEHRTNHCPADGDDDPGLSWVASSPSSKDVASPSQMLGDGCCDMGMGTG

EEEGGSGLPYPCQFCDKSFSRLSYLKRHEQIHSDKLPFKCTFCSRLFKHKRSRDRHVKLHTGDKKYSCQE

CEAAFSRSDHLKIHLKTHSSSKPFKCSICKRGFSSTSSLQSHMQAHRKNKEHLAKKDQGKRDGSSSDVTE

QDQDLYMCDYCEETFSQTDELEKHVLTQHPQLSDRAELQCIHCPEIFSDEGTLLTHIDRTHANKKHKCPM

CAEQFPSVEDVYCHLDSHRQPDSSNHSASPDPVLGSVASMSSATPDSSASLERGSTPDSTLKPGQSRRKL

APSSDHDDGTWSGKVTYSCPYCSKRDFNSLAVLEIHLKTIHADKPQQSHTCQLCLETLPTLYNLNEHVRK

AHRSSGNSASNFPLLQFSNVSAFHCNYCPDMFADINSLQEHIRVSHCLSGGVVAGSTTLEGNHAFFCNQC

SMGFLTESSLTEHIQQTHCSSVGGVTKMESPVLQPSQSFMEVYSCPYCTNSPIFGSLLKLTKHIKENHKN

IPLANNKRKVKVADLSPASSDVEISSPKRHRVTGDSTPAVANGDYPCNQCDLRFSSFEGFQAHLKSHLEL

LLRRQSCPQCNKEDFDSQEALLQHLTIHYTTTSTQYVCESCDKQFSSVDDLQKHLLDMHTFVLYHCTLCQ

EVFDSKVSIQVHLAVKHSNEKKMYRCTACAWDFRKESDLQLHVKHSHLGHPASTGAPGKARKCIFCGETF

GTEVELQCHITTHSKKYNCRLCGKAFHAIVLLERHLREKHCIFDGGNGNGNGGSQNGTPNGVTQSSKRST

AGSTAAAEQADLQNMLLKGGSQETANSHEASGGEEELDASEPMYACDICGAAYTMESLLQNHRLRDHNIR

PGEDDAGSRKKKADFIKGNHKCNVCSRTFFSENGLREHAQTHRGPAKHYMCPICGERFPSLLTLTEHKVT

HSKSLDTGTCRICKMPLQSEEEFIEHCQMHPDLRNSLTGFRCVVCMQTVTSTLELKIHGTFHMQKLSSSG

GSGGGGGSASSSPNGQLQAHKLYKCALCLKDFKNKQELVKIDVNGLPYGLCAGCMSRGTNGQSPTVVVTP

QEAGDKGTTGLRCSECAVKFETLEDLESHIQVDHTEMSPETSGAKKVTDASPVPKKKTYQCIKCQMTFET

EREIQIHVANHMIEEGINHECKLCNQMFDSPAKLLCHLIEHSFEGMGGTFKCPVCFTVFVQANKLQQHIF

AVHGQEDKIYDCSQCPQKFFFQTELQNHTLSQHAQ

>SeaUrchin ORF generated from strPur1_dna range=Scaffold109473:17367-22190 5'pad=0 3'pad=0 revComp=FALSE strand=? repeatMasking=none

MSIHIQQIHIKYPSTPQSYVCHYCGKEYPSLFNLTEHISALHENEAKNMQSGDEACNFICGYCTMKFSTM

TELHEHVKGVHSMTGLISYREDGKLVCPYCNAAFPSEVALMDHFRNVHNSFDNAKDRSKLQCPHCTKAYP

NERYLQEHMKRSHCKPLDKMKYPCPYCIKQNLFDSIEQLQLHIEVFHKSSHTPVYLNSESTCSSSSGSIL

KDQLSGKSNLDKLSPPPISIPSFKVTKDVKEMRVVKKEPATSPIPAPVSISVSPPEDPEHQSQSSEGSTT

EETKVSCPTCHNEFSNKEQLIVHVLTHFKTVSKEYVCKDCGKSFKKPDELQKHLFEIHAHHLFKCSLCKE

VFDSKVSIQVHFAVKHSNEFKIFSCTKCGVVYNAEAELLNHVKAXXXXXXXXXKCIFCSQSFASDVEFQC

HLTTHSNQLRCPFCNAPYRSQEALDIHLQAAHDHVGGSSTLAKPDGPAETPEEVHGRTSVDSAKKSSIKS

EGSAKSPKTKEDIVTDEVAGCFTCQICDVMFPLKSLLEKHREQEHNIKARSATIKMEMTQSINIADHIKG

KFSCQICPEKFKYKFEMMKHLNDHSMQEKQTLAQIARSKSPAMSPRSASPQQPCVCQVCHQSLRSEAEFL

DHTQRHNVDLSLGSERIRCLVCLQELTSLVELQLHARHHTQIVSSLSGQELYPCYLCGKAFASRADVVPK

LDSDGRPCFSCMRCVKTTLEAQFHGVTKLGQSMNQLGGQFSKSNGEMTPDSIVAVHRCTVCRVKFESQEE

LVIHMQVHSQSTSNREANTSAVGSSTGKTYQCIKCQKTFATEAEIQLHVTSHVLAEGVLHECKLCRQVFD

SPAKLQCHLIEHSYPDKEYRCPICRALFSCANDIQAHAIEHGMDSRHHKCMNCNQSFFFPAELMNHAKSH

PQCHQESKFNCGECKQSFASLFSLSNHMKLHLNKPTVKCSVCPEVFQSVLEMQHHYFRVHSEAEVVEKPK

TYDCTKCGKNFPCLSNLQGHMRIHHEGKKYTCGECNKVFALARNLTIHMRSHSGEKPYQCPICDKRFARK

ENRKVHMQSHSGVRPFMCPHCGKMFSRKFHVQVHMRTHSKSRMTSTHQCEICQDNFTLAKNLRRHLKKVH

KMAHHASSSGMVVEYPDGDMKSKRPSVLDVQESSNHSIKIEPSSSSQPNSQSGTPDTSLNCAGWS

>Drosophila|gi|20129991|ref|NP_610967.1| CG17390-PA [Drosophila melanogaster]

MMALKMLYRGPSSRLENLIEKIQATKEITNNDMYSTHTSSSYSPSISDGTMTPNSHHLIGAPTAAGQEDH

PTEGKINGGADGEDLPKPKRLPHFHHHHHHHYHHQQALKIANKLRKINKEAKMGATAGGGATGAASKFDK

LTGEGIKSRGDGSYQCQFCEKTFPRLGYLKHHVQVSYQIEWVQSSKELQSRKGLPSSDLYRPPGSNMLRS

GFDPSAWMGWYGSEFGLSAYGMLMAMSHAEHLPFKCEYCSKLFKHKRSRDRHKKLHTNERNYKCPHCEAA

FSRSDHLKIHMKTHDIQKPFQCSMCNRGYNTAAALTSHMQKHKKNAAILAAGGNPNALNYSPRSTGSASA

SVSSNGSLQKRRYALALASDSSPSRMDFPKRSRSNHVGGTTTTATPTPLLRCSYCPKVTEFSSLEQLNAH

LQSVHEQPQTQAVKTPVQEGEGFQLSCEYCTMKFGNIAGLFQHMRSTHMDRLSSPNSYYEHFNRLATAGT

FSPRLALDLPKIKPDLGSPERESRPAEDDLPTDLSNNKRRPLTPNPQAQTPLAPPSAPPGVFFCNQCNAG

LPDFESFRNHLKSHIAEGMQLVCPHCGMSLPEQSEFERHVVGHFLITGSEFNCSSSCGKSFAKSEDLQQH

LLSEHVLTLLKCSLCSELCESRMAMQLHLACAHSQETKLLRCSACLELFRSDAEFHVHVKTRHQLGGHPT

LGATSSAPTNPLQCMFCRAVCSSELEMHFHLAAHARQFRCPSCPETFHVEFLLDRHMQSQHGGVKDKEAN

SPNMGSLYVNALLPPLAAAAAAAAATNNNSSIIDYNVAFKGLFGGASGGAGSGGGGAQSGGAPPSANKFY

SPLQVDTNALKAQTSPHPALMYGLSQRYLMEMYAAKSTSPSGNEGVGNSQPPAPQATAPPPPPNASTATF

SCGMCERQDLRSEAELHSHRKLAHNLKTGVSLRCAYCAGNFKSRAELEQHMKSCHNSTGKHKCLICDEVF

PSPAILAEHKLQHSKVGQSGKCSHCGQPLEDVAAFRAHLSEHGSDGASLPLACICCRQTLHSEFELSLHA

KFHTKSSSSGGSLQEPVCALCLEPLPDATEGPAKLCDKCCRKHNLNGKRGKHSEPATSLPAPPSAFVENR

CNLCKMILPHAQKLQEHLVEHTFAGTEQRGFNCYICSAVFTAPGGLLNHMGEHGAHSRPYDCNLCPEKFF

FRAELEHHQRGHELRPQARPPAAKVEVPSIRNTSPGQSPVRSPTIVKQELYETDTVESAGVEDEPENHPD

EEEYIEVEQMPHETRPSGIGSQLERSTSSA

>Honeybee|gi|110762676|ref|XP_396910.3| PREDICTED: similar to CG17390-PA [Apis mellifera]

MLFKGNSSRLELLIGKIQAHKEPSQEEQQKSKEALGTGSSSWQSEDGGPNSRRGGETPSSCATPTSASFP

SEPEVDADVGVNADGSNATAPYPCQFCDRTFPRLSYLKKHEQVSIQEDYYYYYYYRYVYSFLLMSQSHGD

QMPYRCSWCARLFKHKRSRDRHVKLHTGDRRYRCTHCEAAFSRSDHLKIHMKTHDTQKPYQCTACSRGYN

TAAALTSHMQSHKKHHQSQGSTKDIDYGRRSVSSHSTSSPPVPSSPSPSLNLALNPKPGLKSSQGSASTT

PILNSPLKLACMYCTRDSFNCMQQLQMHVHTMHQAILSGETVAVSPSTNRGSEPIGYQHGEKNSDRSEGT

SKEHRKYREDDSERSMEKDHYENAFTCNQCTMKFSTLGSLRDHLISIHRTDSFNSALMMCPLCGIPCASA

AAYAEHYVLQHCENRSIGPLESKDYMDAKMNGNYESKSSRNQKCREQISCSAEPADLTSKHTATTENNYS

AGTLLCGQCGAALKDFESFREHLARHLQANHRNDAVRHSCPKCEATFQDREDMLVHLTKHYLGQISKEYA

CGACKKLYPHPDLLQRHLLDSHAHHLYRCALCKDTFDSRVAIQVHFAVKHSQECRIYRCNACTVSNNENS

PGNAPGEGKSFFRSESEMTNHVKNVHAPPTVSNNSPVARSPASTPGITGNSGPRCVFCGICCTSELELQL

HLASHSVNLYRCPVCREGFAVEFLLDRHIAQAHHSSNHQGIIRSSSRENGRIAHHSSNHQGIIRSSSREN

GRIGRPPRLQEETKSQKRGRSPASSNNNTVNQRDNNNKRPNYSNTSSQQCDLCERGEFSNETELQAHKKL

VHTPAKFQNKSLSNLSMTCAYCGEVCRSRTDLESHTRIQHASNEPGGRHKCNICDEVCPSGATLAEHKLQ

KHCKIQLSDTCIVCRGNLASESQFLEHVQRHSLENVDPQQRLDGSLPHLPAACVVCRQTLISDLECRLHA

RHHLRASTGAHSVGSSPSPNQKNQNPSCCLCLRDFSSDDFVNLPPSHVSGGGQSLRVCKSCYIRHSQGLP

ILNSYESVRSKCDDSWTSNNKDGQWDGSKNKWESENKYKENRNGMIDNKRCQDCGVKFEDSEEVEKHRII

EHEKTGTASNTYTCIQCQMSFATEAKIQQHIKKEHLEVAGKTSMEALRCHLCLFEANSPLQLQSHLIEHT

FAGCAALSCYICQSLFTAPIGLQNHMLQEHGLGARPYDCSQCTLKFFFRAELDHHILTFHRSGDVSSPDE

DIQNNIETKPRDTEENNCDERIMVKEELLPEAADEEEEINVDEQVEENEQKEDKQLEFETKLKTEIEEEN

ILQSSISEKMES

>Anopheles|gi|116131769|gb|EAA05280.3| ENSANGP00000003937 [Anopheles gambiae str. PEST]

MALKMLYRGPSTRLETLIEKIQQNTNAFELYSSHTSSSFSPSISDGMTTPNSIPEPNDGPTTIDSGAAGE

KQYRNHHPHHQLHPRHVHHLHHRRAGGIGGHSSTGGKSSSKHHQQHNNNNSNNHHNHNNNNNGKGLGGGA

GSSTYHCQFCEKTFPRLGYLKKHEQVRDGLLYKTEQNCEQRRRSVQKAAHQTMRGMTIFAAAAAVGRFMS

YVYSIRFAKIERGRESKSHTEHMPFKCEYCARLFKHKRSRDRHTKLHTGDRRYRCLHCEAAFSRSDHLKI

HMKTHDNQKPFQCTICNRGYNTAAALTSHMQNHKKQLALTGSPNLTYSPRSTTSSLSSGGGSGGGGKRGA

KFSPYSNDPLMLMSRGNSKLGAGGGTGTSHHQQDLLSCPYCTRTDFGTLEQLGLHVQSMHGHVTPDGSNV

PLSLGGLSPYPPISCEFCTMKFPTVPLMFAHLKTAHLDRLAGSGGGHQRSSPSNGTGSPLGSRPPSGGRA

GSHPSGRTSPAEQLTPTDLSQPKQMGAPNSSGSSSQHHHQPPGAYLCNQCNAALPDFESFRTHLKAHLEQ

SAASAAAAAAMRLCQAEYEQHTIGHFLVMAVEYRCQGCSGKSTFGKVEDLHKHLYEGHMQLLYKCTVCGE

MFESKVQVQVHFAVSHSVEVKLYRCSACAEVFRSERDFRQHIRNRHLTAGAVQCMFCRMVCSSELEMHFH

LASHARKYKCPACPESFHVEFLLDRHIQMHHSQKETSSPNRGRESANGGHNLSTTSSCSTNSTPSTATAV

SANATANALLGLGYGGASLTSGNSQQPRTSTLNGSSSGLYSPEEGPNGGSSVATSSSKLYSPIAMIQRSL

PEHHPEQQQSSNRKTAQQQSGPKTSSGTITPQLIAPGSNSCYSCGICERSDFSTESEVQTHRKIVHNLKT

GVSLRCAYCNGDFRSRNELENHMKVAHNTGGGKHKCLICDEIFPSPAVLAEHKLTHCKVGASGRCSHCSL

PLPDAHTFKQHLPAHQQIECDRFPQQCICCRQTLNSEFEISLHAKFHTKTAETNERTCALCLEPLPSQLE

SNNTKICDPCLKRHNFPTKLLSMNFLKPATPSLVPTPTHGSSSALFQCNLCKKPLPSAQKLQEHLIDHTF

AGCEERGYVCYLCSAVFTSSAGLQTHLPLAHSNAAAKPYDCERCGVAYFFRAELEHHLIDHELGKAIRPS

SFEV

>Beetle|gi|91075920|ref|XP_966615.1| PREDICTED: similar to CG17390-PA [Tribolium castaneum]

MKMLFKGNSSRLELLIEKIQSTKEGEFKVPVAEDETAASSASGGSGCGETQPFDDEQVQGDQDQDTKPGP

YKCTVCGENYLKEKLLREHEQSHTDHLQFTCAYCPRLFKHKRSRDRHTKLHTGDKKYKCQQCDSAFSRSD

HLKIHMKTHDSRKPYKCGTCNRGYNTAAALSSHQQSHLKQESRSGSRTSGGSTPSPGLFRCTHCAETFGK

PDLLQSHVAMVHSDTDSSLSQTPEPLSEYQNQIEDLKIVCMYCSKEFPSLELMYQHTNIAHRDVPNGVTT

SVSVSSPAIQNSPKPEICQNGTPTYACDRCTMQLDSLQNLKNHINNVHWRPALSPNPVNFMGIEKSYSPF

QTQPTDLSRKKKSEDSAEKAVKKKKDQHSPTAISPYDSNDKPCICSCCYAQLPNFKSFLHHMESHVISSN

NSLLGFCPVCGEPGRDPVSFTNHIFSHAIAQVPGRCCYTCKKSFERLEELQKHLLEVHVVSVFKCSICND

IFDTKISMQLHLTNKHSDECKHFKCYLCTNQVFHDRLSAELHISMKHYQQFTPCVGANQLLRSQYQELDT

RFRDYNMIFQCTFCHKTFKDQYSQYIHILKEHNESKEGEKFILDSAMNPNPSRSPNFMFPKIVTPDHVEP

LESVYTCDICNRSDITSESDLINHKKLHHSSKNKIGPVSLQCAYCNEYCKSRTDLENHMKTHQVTCGKGK

HKCNICDEIYSSTLTLADHKLTHCKIVEGNSCVQCKSVLTDENSFYSHQLQHSNPGKPNSQISLPANCII

CCQTLQTDVEIKLHAKFHLKHLTQKEYMCGVCSKVFDSQGQPANDVNIVVCKDCAKSEDEGKKVPKQFAC

IQCPQTFDSESDVQNHAAMHMLNEGTNLECRLCKQVFSSPLKLQTHLIEHNFYGMNQYSCYVCSSVFTAA

SGLQSHIIGHGLDSRPYECSQCQMKFFFRAELDNHQRGRDLREQAKPPCPFRISPKRETFDNEVDNNIKQ

EVTEIASEEKDEQ

>Xla423|gi|262050511|ref|NP_001159911.1| zinc finger protein 423 [Xenopus laevis]

MSRRKQAKPRSVKVEDAEDFGLNWESTVNQTGGLDRETDGEGQTLEDGNSMTSQEERIEEEELEDESIYT CDNCQQDFDSLAELTEHRTQHCLGDGDDDPQFSWVPSSPSSKDVASPTPIIGDGCDLGIGEEEGGSGLPY PCQFCDKSFSRLSYLKRHEQIHSDKLPFKCTYCSRLFKHKRSRDRHIKLHTGDKKYHCHECEAAFSRSDH LKIHLKTHSSSKPFKCTVCKRGFSSTSSLQSHMQAHRKNKEYMTKSDKEMKKDDFMCDYCEETFSQTEEL EKHVMTRHPQLSEKADLQCIHCPEVFADESSLLTHIDQVHANKKHKCPMCPEQFSSVEEVYCHLDSHRQP DSSNHSISPDPVLGSVASMSSATPDSSASLERASTPDSTLKPIRGHKQTGSLEREEGQNWPKIAYSCPYC SKRDFNSLAVLEIHLKTIHVDKPQQNHTCQICLDSLPTLYNLNEHVRKVHKNHAYPMVQFSNITAFHCNY CPEMFADINSLQEHIRITHCGPNATPQEGNNAFFCNQCSMGFLTESSLTEHIQQTHCNVGSSKLESPVIQ PTQSFMEVYSCPYCTNSPIFGSILKLTKHIKENHKNIPLANHKKSKSEQSPVSSDVEVSSPKRQRLCASL NSVSNGEYPCNQCDLKFSNFDTFQTHLKSHLELLLRKQSCPQCKEDFDSQESLLQHLTIHYMTTSTHYVC ESCDKQFSSVDDLQKHLLDMHTFVLYHCTLCQEVFDSKVSIQVHLAVKHSNEKKMYRCTACNWDFRREVD LQLHVKHSHLGNPSKSHKCIFCGETFSTEVELQCHITTHSKKYNCKFCSKAFHAIILLEKHLREKHCVFD TNNQNGTANGMVPSNKKSESVEAQSGLMKNPDVTNSHDASEDDVEASEPMYGCDICGAAYTMEVLLQNHR LRDHNIRPGEDDGSRKKAEFIKGTHKCNICSRTFFSENGLREHMQTHRGPAKHYMCPICGERFPSLLTLT EHKVTHSKSLDTGTCRICKMPLQSEEEFIEHCQMHPDLRNSLTGFRCVVCMQTVTSTLELKIHGTFHMQK LAGNSAASSPNGQTLQKMYKCAICLKEFRNKQDLVKLDVNGLPYGLCAGCLSRSTNGQPSNPTPQEACDR LCTSLRCSECSVKFESTEDLETHIQIDHRDLAHDGSGQRKVAQSSPVPRKKTYQCIKCQMTFENEREIQI HVANHMIEEGINHECKLCNQMFDSPAKLLCHLIEHSFEGMGGTFKCPVCFTVFVQANKLQQHIFAVHGQE DKIYDCSQCPQKFFFQTELQNHTLSQHAQ


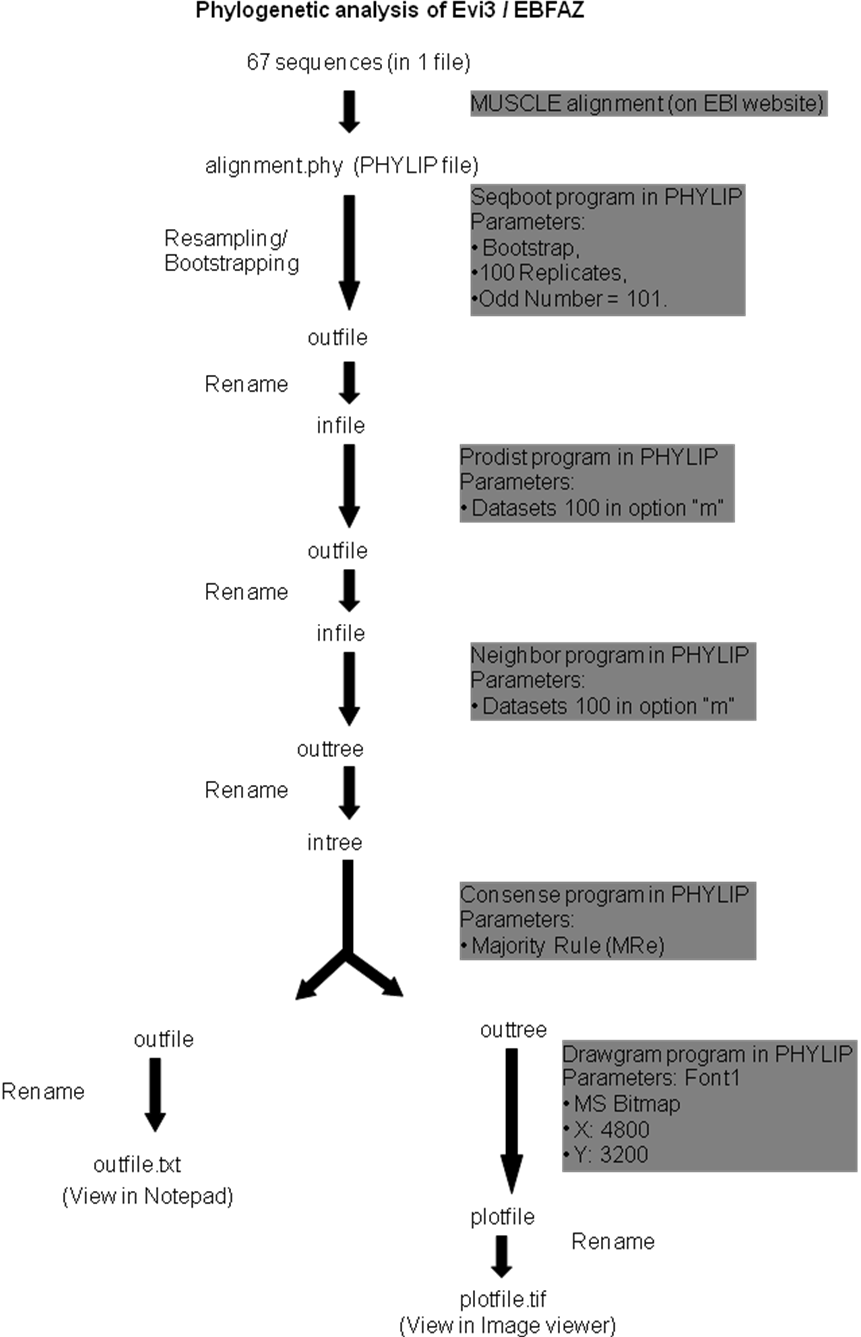

Supplement: Supplementary file 2 — Supporting Information Table 1 [file DVG-54-519-s002.doc]
