# Supplementary material for: Transcriptional regulation of the proto‐oncogene Zfp521 by SPI1 (PU.1) and HOXC13
Source: Genesis. 2016 Aug 29;54(10):519–33. doi: 10.1002/dvg.22963 (PMC5073027; doi:10.1002/dvg.22963)
Supplement: Supplementary file 3 — Supporting Information Table 2 [file DVG-54-519-s003.doc]

Supplemental Table 2. Primer sequences.

| Primer set | Forward | Reverse |
| --- | --- | --- |
| Human *ZNF521* qPCR primers | CCACATCCAAACCATCCACCG | CAGGTGGCACTGGAGTTTGGC |
| Human *HOXC13* qPCR primers | AAGGTGGTCAGCAAATCGAAAG | TGGTACAAAGCGGAGACATAAATAGA |
| Human *SPI1* qPCR primers | CAGCTCTACCGCCACATGGA | TAGGAGACCTGGTGGCCAAGA |
| Human *GAPDH* qPCR primers | ACCATCTTCCAGGAGCGAG | TCACGCCACAGTTTCCCGGA |
| *Zfp521* promoter 0.2Kb forward | gcc tcc agt ccg ggt ctt |  |
| *Zfp521* promoter 0.5Kb forward | ttc tac cag aag ggg gac ct |  |
| *Zfp521* promoter 1Kb forward | cgt tta aaa act att ttc tta tcc aga |  |
| *Zfp521* promoter common reverse primer |  | aat gga aaa tcc aag caa gg |
| SPI1 ETS-domain truncation mutant | ggggagacaggcagctagaaaaagattcgcc | ggcgaatctttttctagctgcctgtctcccc |
| *Zfp521* promoter IP primers | CCCGACTGGGCGACTGAAGC | GCCCCTAACCCGCAGGGACT |
| *Hoxc13* RT-PCR and qPCR primers | ACAGTCAGGTGTACTGCTCCA | TCGATTCTGGAACCAGATGGT |
| Mouse *Spi1* RT-PCR primers | TTTCCTACATGCCCCGGATGT | GGTAGGTGAGCTTCTTCTTGA |
| *Gapdh* mouse qPCR primer sequences | AACTCGGCCCCCAACACT | TCTAGGCCCCTCCTGTTATTATG |
| *GFP* primers | GACACCCTGGTGAACCGCAT | TGCCGTCCTCGATGTTGTGG |

EMSA binding site sequences

| Promoter binding site | Oligo 1 | Oligo 2 |
| --- | --- | --- |
| HoxC13 binding site | TCACTAGAGACACC**TCATTA**  **A**TCTGTTTGGTAAC | GTTAGCAAACAG**ATTAATGA**GGT  GTCTCTAGTGA |
| HoxC13 Mut binding site | TCACTAGAGACA**CCTCCGGCA**T  CTGTTTGCTAAC | GTTAGCAAACAGA**TGCCGGAG**GT  GTCTCTAGTGA |
| SPI1a binding site | CTTTAATTTCTGGA**GAGGAA**GA | TC**TTCCTC**TCCAGAAATTAAAG |
| SPI1b binding site | TGACAATGAT**TTCCTC**AGTGATTA | TAATCACT**GAGGAA**ATCATTGTCA |
| SPI1a Mut binding site | CTTTAATTTCTGGA**ACACTA**GA | TC**TAGTGT**TCCAGAAATTAAAG |
| SPI1b Mut binding site | TGACAATGAT**ACACTA**AGTGATTA | TAATCACT**TAGTGT**ATCATTGTCA |
